# Synthesis, characterization, and computational evaluation of some synthesized xanthone derivatives: focus on kinase target network and biomedical properties

**DOI:** 10.3389/fphar.2024.1511627

**Published:** 2025-01-03

**Authors:** Wisam Taher Muslim, Layth Jasim Mohammad, Munaf M. Naji, Isaac Karimi, Matheel D. Al-Sabti, Majid Jabir, Mazin A. A. Najm, Helgi B. Schiöth

**Affiliations:** ^1^ Department of Pharmaceutical Chemistry, College of Pharmacy, Kufa University, Najaf City, Iraq; ^2^ Department of Microbiology, College of Medicine, Babylon University, Hilla City, Iraq; ^3^ Clinical Laboratory Sciences, College of Pharmacy, Kufa University, Najaf City, Iraq; ^4^ Research Group of Bioengineering and Biotechnology, Laboratory for Computational Physiology, Department of Biology, Faculty of Science, Razi University, Kermanshah, Iran; ^5^ Department of Science, College of Science, Uruk University, Baghdad, Iraq; ^6^ Department of Applied Science, University of Technology, Baghdad, Iraq; ^7^ Department of Pharmacy, Mazaya University Collage, Nasiriyah, Iraq; ^8^ Department of Surgical Sciences, Functional Pharmacology and Neuroscience, Uppsala University, Uppsala, Sweden

**Keywords:** xanthone, total synthesis, ADMET, molecular docking, network analysis, target fishing

## Abstract

**Background:**

Xanthones are dubbed as putative lead-like molecules for cancer drug design and discovery. This study was aimed at the synthesis, characterization, and *in silico* target fishing of novel xanthone derivatives.

**Methods:**

The products of reactions of xanthydrol with urea, thiourea, and thiosemicarbazide reacted with α-haloketones to prepare the thiazolone compounds. Xanthydrol reacted sequentially with ethyl chloroacetate, hydrazine, carbon disulfide, and α-haloketones to prepare the dithiolane. The xanthydrol reacted with propargyl bromide and it submitted to click reaction with azide to prepare triazole ring.

**Results:**

Finally, four novel xanthones derivatives including (*E*)-2-(2-(9*H*-xanthen-9-yl)hydrazono)-1,3-dithiolan-4-one (L3), 2-(2-(9*H*-xanthen-9-yl)hydrazinyl)thiazol-5(4*H*)-one (L5), 2-(9*H*-xanthen-9-ylamino)thiazol-5(4H)-one (L7), and 4-((9*H*-xanthen-9-yloxy)methyl)-1-(4-nitrophenyl)-1*H*-1,2,3-triazole (L9) were synthesized and characterized using thin layer chromatography, Fourier-transform infrared spectroscopy, and nuclear magnetic resonance (^1^H and ^13^C). ADMET, Pfizer filter, adverse drug reaction, toxicity, antitarget interaction profiles, target fishing, kinase target screening, molecular docking validation, and protein and gene network analysis were computed for derivatives. Ligands obeyed Pfizer filter for drug-likeness, while all ligands were categorized as toxic chemicals. Major targets of all ligands were predicted to be kinases including Haspin, WEE2, and PIM3. Mitogen-activated protein kinase 1 was the *hub gene* of target kinase network of all derivatives. All the ligands were predicted to show hepatotoxic potentials, while L7 presented cardiac toxicity.

**Conclusion:**

Acute leukemic T-cells were one of the top predicted tumor cell lines for these ligands. The possible antileukemic effects of synthesized xanthone derivatives are potentially very interesting and warrant further studies.

## Highlights

• Xanthone derivatives possess numerous significant properties with biomedical applications.
• Four xanthone derivatives were synthesized from xanthydrol using dithiolan and thiazol.
• All stable xanthone derivatives were characterized using TLC and FTRI, and they were stable.
• Ligands did not express any violations to the Lipinski (Pfizer) filter for drug-likeness.
• Major targets of xanthone derivatives were subsets of the kinase family.
• Among synthesized derivatives, L9 expressed promiscuous potential in target binding.

## 1 Introduction

Xanthydrol (C_13_H_10_O_2_; total molecular weight of 198.221 g/mol; Supplementary Figure 1) belongs to heterocyclic compounds known as xanthones or 9H-xanthen-9-ones, characterized by a tricyclic framework containing two benzene rings fused to a central pyrone ring. This heterocyclic system is widely in nature and is found in various natural products and bioactive compounds (Goldberg and Wragg, 1957; Badiali et al., 2023). Xanthydrol-based assays offer a sensitive and reliable method for measuring urea concentrations, providing valuable information for diagnosing and monitoring conditions such as kidney disease and dehydration (Supplementary Scheme 1) (Abrahamson, 1956). Xanthine and its derivatives have anticancer, anti-inflammatory, and antioxidative effects (Huang et al., 2021; Ramakrishnan et al., 2021; Kapri et al., 2022; Veríssimo et al., 2022). Moreover, recent studies have focused on innovative chemical synthesis approaches involving xanthydrol derivatives to develop novel compounds with potential anticancer properties. These studies aimed to harness the inherent reactivity and structural characteristics of xanthydrol for the targeted synthesis of compounds that could exhibit enhanced efficacy against cancer (Jiang et al., 2018). Synthesis and biopharmaceutical evaluation of xanthone derivatives were pursued by some researchers because these chemicals exert good anti-cancer activity by targeting topoisomerase II (TOP2) (Song et al., 2022). Besides their therapeutic potential, xanthydrol derivatives have been employed as fluorescent probes for cancer imaging, enabling researchers to visualize and study specific cellular processes associated with cancer. The development of these probes allows for precise and real-time monitoring of cancer-related biomolecules, contributing to the understanding of tumor progression, and facilitating early detection (Liu et al., 2018; Tian et al., 2024). In summary, further investigations are warranted to determine the targets and probable promiscuity potential of these derivatives.

Xanthones have emerged as a promising source for drug development especially in oncology (Kurniawan et al., 2021). Xanthones are a class of three-ring phenolic acids with a wide range of bioactivities, including antimicrobial, antiviral, anticancer, antioxidative, antidiabetic, and anti-inflammatory effects (Choodej et al., 2022). For instance, gambogic acid, a prenyl xanthone known in *Garcinia hanburyi*, Clusiaceae, exhibited noteworthy induction of apoptosis, inhibition of cell proliferation and tumor angiogenesis, and antioxidative and anti-inflammatory activities (Wang et al., 2009; Chen and Chen, 2012). In this line, xanthones exerted antitumor effects mediating through autophagy, apoptosis, cell cycle arrest, and tuning signaling pathways such as PI3K/Akt and MAPK (Gunter et al., 2023). More interestingly, in a second-phase clinical trial conducted in China, *algambog acid*, a derivative of xanthones, was investigated for its anti-tumor properties (Zhou and Wang, 2007). Furthermore, *gambogic acid*, a xanthone found in mangosteen, is dubbed as a dietary supplement for immune promotion, anti-inflammation, and induction of cell-mediated immunity (Gutierrez-Orozco and Failla, 2013; Rizaldy et al., 2021). In this continuum, a comprehensive survey has summarized the structure-activity relationships (SARs) of xanthones, providing valuable insights for further drug research, expansion, and development (Miladiyah et al., 2018). For instance, triazoles as pharmacophores are five-membered heterocyclic rings containing three nitrogen atoms with two main types of 1,2,3-triazoles and 1,2,4-triazoles (Supplementary Figure 2) (Matin et al., 2022; Raman et al., 2024). Both triazoles have tautomerized as reported previously (Supplementary Scheme 2) (Pylypenko et al., 2023). Numerous 1,2,3-triazoles have been synthesized through 1,3-dipolar cycloaddition reactions of acetylenes with azides. Typically, more electron-withdrawing substituents on the acetylene facilitates the cycloaddition reactions. Conversely, electron-withdrawing substituents on azides exert the opposite effect. Bulky substituents on azides may hinder the reaction rate but often result in an improved selectivity (Supplementary Scheme 3) (Dai et al., 2022; Drelinkiewicz and Whitby, 2022). Therefore, xanthones and their derivatives are promising anticancer compounds and they can be submitted to the computational pipeline of drug discovery and design.

Dithiolane (C3H6S2) is a diverse organic cyclic compound comprised a pentagon ring containing two sulfur atoms, giving rise to different structural isomers, including 1,2-dithiolane and 1,3-dithiolane (Supplementary Figure 3). Derivatives of this compound are collectively referred to as dithiolanes (Taguchi et al., 1987). Dithiolane compounds, exemplified by dithiolane and its derivatives, have emerged as intriguing subjects in cancer research due to their potential therapeutic effects (Nikitjuka et al., 2023). Dithiolan compounds have emerged as intriguing candidates in cancer research, offering potential therapeutic avenues for combating various malignancies. These compounds were derived from a cyclic structure saturated with chemicals and they have diverse pharmacological properties in the cancer progression (Cao et al., 2023). In addition, their antioxidative activity may help counteract oxidative stress, a factor linked to cancer development (Theodosis-Nobelos et al., 2021). Also, dithiolanes have shown antiproliferative effects by influencing cell cycle progression and inducing apoptosis, providing potential avenues for inhibiting cancer cell growth (Ismail et al., 2023). Mechanistically, dithiolanes may interfere with crucial mechanisms involved in cancer progression through modulation of PI3K/AKT/mTOR and MAPK pathways (Felber et al., 2023). Furthermore, certain dithiolane compounds have been explored for their ability to enhance cancer cell sensitivity to chemotherapy, potentially improving treatment outcomes (Nikitjuka et al., 2023). Dithiolane was initially prepared by reacting carbon disulfide with methylene-active compounds in the presence of sodium ethoxide, followed by adding 1,2-dichloroalkanes to the reaction mixture (Supplementary Scheme 4) (Greene and Wuts, 1999). In conclusion, dithiolanes are pharmacophores used for anticancer drug design.

Thiazole (C3H3NS), also known as 1,3-thiazole, is a heterocyclic compound containing sulfur and nitrogen atoms, existing as a pale-yellow liquid with a pyridine-like odor (Supplementary Figure 4). Its structural simplicity contradicts its importance, as the thiazole ring serves as a component in the various biologically active molecules including vitamin thiamine (B1) (Kashyap et al., 2012). Although studies focusing on the direct roles of thiazole in oncotherapy are scarce, its derivatives showed promise in combating cancer through multiple mechanisms, including antiproliferative effects, induction of apoptosis, and inhibition of angiogenesis (Elmaati and El-Taweel, 2002; Mohareb et al., 2013). Thiazoles illustrate extensive applications in various fields due to their diverse properties. It is widely utilized in decomposing agricultural chemicals and pharmaceuticals, acting as a precursor for non-peptide protease inhibitors of human immunodeficiency viruses and anti-schistosomal agents (Cascioferro et al., 2020; Ibrahim and Rizk, 2020). Moreover, thiazole plays a crucial role in the production of tinctures in pharmaceuticals, and thiazole derivatives have demonstrated anticancer activities through various portals (El-Taweel and Elmaati, 2002; Shawali et al., 2002; Abdelrazek et al., 2010). Therefore, development of novel synthesized xanthone derivatives using thiazoles may have large potential for applications in both industry and medicine.

Here, we initially reported some synthetic and physicochemical features of xanthones derivatives and finally, we used a package of *in silico* tools to predict toxic and pharmacological properties and targets of xanthones derivatives for future applications in oncotherapy.

## 2 Experimental section

### 2.1 Chemistry

All reagents used in the synthesis and characteristics were purchased from Sigma-Aldrich Co. and Merck Co. (United States) and used without additional purification. Melting points were determined with a Thomas–Hoover capillary apparatus (Thomas Scientific, United States). Perkin Elmer Model 1,420 spectrometer was used to acquire infrared spectra (Thermo Fisher Scientific, United States). ^1^H-NMR spectra with tetramethylsilane (TMS) as an internal standard were acquired with Bruker FT-500 MHz instrument (Brucker Biosciences, United States). Chloroform-D was used as a solvent. Coupling constant (J) values were estimated in hertz (Hz) and spin multiples were given as s (singlet), d (doublet), t (triplet), q (quartet), and m (multiplet). The mass spectral measurements were performed on a 6410 Agilent liquid chromatography/mass spectrometry (LCMS) triple quadrupole mass spectrometer (LCMS; AGILENT Technologies, United States) with an electrospray ionization (ESI) interface. Microanalyses, determined for C and H, were within ±0.4% of theoretical values. Analytical thin layer chromatography (TLC; CAMAG, Switzerland) was performed on silica gel plates using a mixture of ethanol in different proportions, coloring agents, benzene, and methanol to obtain different polarity and the best retention factor readout.

### 2.2 Synthesis

Many heterocyclic compounds were prepared (*vide infra*). In brief, xanthydrol reacted firstly with urea, thiourea, and thiosemicarbazide; then, the products reacted with α-haloketones to prepare the thiazolone compounds. Secondly, xanthydrol reacted firstly with hydrazine; then, it reacted with carbon disulfide. The resulting products then reacted with α-haloketones to prepare dithiolane. Thirdly, xanthydrol reacted with carbon disulfide and then reacted with α-haloketones. Fourthly, xanthydrol reacted sequentially with ethyl chloroacetate, hydrazine, carbon disulfide, and α-haloketones to prepare the dithiolane. Fifthly, xanthydrol reacted with propargyl bromide and it submitted to click reaction with azide to prepare a triazole ring.

#### 2.2.1 Synthesis of (9H-xanthen-9-yl)hydrazine (L1)

A mixture of xanthydrol (0.01 M, 2.00 g) and hydrazine (0.01 M, 0.504 g) was dissolved in ethanol (25 mL) with a few drops of glacial acetic acid and stirred in a water bath at 67°C for 5–6 h. The progress of the reaction was monitored by TLC. At room temperature (RT), ethanol was evaporated, and the compound was then purified by recrystallization to obtain a whitish powder (Supplementary Scheme 5) (De Perre and McCord, 2011).

#### 2.2.2 Synthesis of potassium 2- 9H-xanthen-9-yl)hydrazinecarbodithoate (L2)

A mixture of compound (L1; 0.005 M, 1.00 g) and CS2 (0.006 M, 0.358 g) was dissolved in ethanol (25 mL), stirred in a water bath at 67°C for 9–10 h, and drops of alcoholic KOH were added slowly. The progress of the reaction was monitored by TLC. At RT, ethanol was evaporated and the compound was then purified by recrystallization to obtain a yellowish powder (Supplementary Scheme 6) (Vasiliev and Polackov, 2000).

#### 2.2.3 Synthesis of (E)-2-(2-(9H-xanthen-9-yl)hydrazono)-1,3-dithiolan-4-one (L3)

A mixture of compound (L2; 0.003 M, 1.00 g) and ethyl 2-chloroacetate (0.003 M, 0.375 g) was dissolved in ethanol (25 mL) using a few drops of tri-ethylamine and stirred in a water bath at 67°C for 6–7 h. The progress of the reaction was monitored by TLC. Ethanol was evaporated at RT, and the compound was then purified by recrystallization to obtain a nutty powder (Supplementary Scheme 7) (Lipin et al., 2019).

#### 2.2.4 Synthesis of 2-(9H-xanthe-9-yl)hydrazinecarbothioamide (L4)

A mixture of xanthydrol (0.01 M, 2.00 g) and thiosemicarbazide (0.005 M, 0.518 g) was dissolved in ethanol (25 mL) using a few drops of glacial acetic acid and stirred in a water bath at 67°C for 5–6 h. The progress of the reaction was trailed by TLC. Ethanol was evaporated at RT to achieve yellow crystals (Supplementary Scheme 8) (De Perre and McCord, 2011).

#### 2.2.5 2-(2-(9H-xanthen-9-yl)hydrazinyl)-1,3-thiazol-5(4H)-one (L5)

A mixture of compound (L4) (0.002 M, 1.00 g) and ethyl 2-chloroacetate (0.003 M, 0.451 g) was dissolved in ethanol (25 mL). The stirring in a water bath at 67°C for 7–8 h. The progress of the reaction was monitored by TLC. Ethanol was evaporated at RT, and the compound was then purified by recrystallization to obtain dark brown crystals (Supplementary Scheme 9) (Zheng and Huang, 2023).

#### 2.2.6 Synthesis of 1-(9H-xanthen-9-yl)thiourea (L6)

A mixture of xanthydrol (0.01 M, 2.00 g) and thiourea (0.005 M, 0.384 g) was dissolved in ethanol (25 mL) with a few drops of glacial acetic acid and stirred in a water bath at 67°C for 6–7 h. The progress of the reaction was monitored by TLC. Ethanol was evaporated at RT, and the compound was then purified by recrystallization to obtain brown powder (Supplementary Scheme 10) (De Perre and McCord, 2011).

#### 2.2.7 Synthesis of 2-(9H-xanthen-9-ylamino)thiazol-5(4H)-one (L7)

A mixture of compound (L6) (0.003 M, 1.00 g) and ethyl 2-chloroacetate (0.003 M, 0.478 g) was dissolved in ethanol (25 mL) and stirred in a water bath at 67°C for 8–9 h. The progress of the reaction was monitored by TLC. Ethanol was evaporated at RT, and the compound was then purified by recrystallization to obtain nutty crystals (Supplementary Scheme 11) (Resende et al., 2020).

#### 2.2.8 Synthesis of 9-(prop-2-ynyloxy)-9H-xanthene (L8)

A mixture of xanthydrol (0.015 M, 3.00 g) and propargyl bromide (0.01 M, 1.200 g) was dissolved in DMF (dimethylformamide; 25 mL) and stirred, then 0.50 g potassium carbonate (K2CO3) was added slowly in a water bath at 75°C for 25–26 h. The progress of the reaction was monitored by using TLC, and it followed by adding 30 mL of distilled water, and two organic and aqueous phases were separated. Finally, the organic phase was evaporated and the compound was purified by recrystallization to reach a dark nutty powder (Supplementary Scheme 12) (Ramakrishnan et al., 2021).

#### 2.2.9 Synthesis of 4-((9H-xanthen-9-yloxy)methyl)-1-(4-nitrophenyl)-1H-1,2,3-triazole (L9)

A mixture of compound (L8) (0.004 M, 1.00) and 1-azido-4-nitrobenzene (0.004 M, 0.731 g) was dissolved in DMF (25 mL) and stirred. Then, 5 mL of the resulting mixture was strongly added to the suspension of sodium ascorbate (0.4 g) and CuSO4.5H2O (0.3 g) in DMF (4 mL) in a water bath at 75°C for 45–50 h. The progress of the reaction was monitored by TLC. The reaction mixture was poured into distilled water (30 mL) and the product recrystallized from ethanol to reach a dark brown powder (Supplementary Scheme 13) (Ghasemi et al., 2019).

### 2.3 Computational methods (*in silico*)

#### 2.3.1 Cheminformatics

The structures of the synthesized ligands were drawn using ChemSketch freeware, and their canonical SMILES formats were submitted to SwissADME (http://www.swissadme.ch/) to compute standard features of drug-likeness. In essence, the calculation of physicochemical properties, medicinal chemistry, and ADMET (absorption, distribution, metabolism, excretion, and toxicity) features using various filters provide information for the estimation of lead- and drug-likeness of hits (Lagorce et al., 2017). For instance, Lipinski’s rule of five (RO5) or Pfizer filter screens lead-likeness of hits based on the criteria including, molecular weight ≤500, MLOGP ≤4.15, the number of (N + O) ≤ 10, and the number of (NH + OH) ≤ 5 (Lipinski et al., 2012; Kralj et al., 2023).

##### 2.3.1.1 Adverse drug reaction (ADR) assay

ADVERPred server was launched at PASS (Prediction of Activity Spectra for Substances) technology (https://www.way2drug.com/PASSonline) for *in silico* prediction of ADRS such as myocardial infarction, arrhythmia, cardiac failure, myocardial infarction, hepatotoxicity, and nephrotoxicity. Prediction is based on the training sets encompassing manually curated information from drug labels. Practically, SMILES formats of synthesized ligands were submitted to the ADVERPred server containing training data sets for similarity research to estimate ADR (Ivanov et al., 2018). In addition, the Toxtree version 3.1.0.1851 software platform (http://toxtree.sourceforge.net/) was hired to forecast the toxicity class of synthesized ligands.

##### 2.3.1.2 Prediction of antitarget interaction profiles

GUSAR software builds quantitative structure–activity relationship (QSAR) models using training sets characterized as structural data files (SDF; Way2Drug.com, 2011–2016). The QSAR models for the sets of 32 endpoints (50% inhibitory concentration—IC_50_, inhibition constant—K_i_, and activation constant—K_act_) comprise the data about 4,000 chemical compounds interacting with 18 antitarget proteins (13 receptors, 2 enzymes, and 3 transporters). The biological profiles for antitargets of our new chemical entities (NCEs) were screened *in silico* using the machine learning-based software PASS 2020 (*vide supra*). The biological profile is categorized as a list of activities along with the probabilities of being active Pa and inactive Pi and with two conditions Pa > Pi and Pa ≥0.5.

##### 2.3.1.3 Target fishing

The SwissTargetPrediction web server predicts probable protein targets based on the structural similarity of NCEs to already known active library compounds. The SMILES strings of NCEs served as input for the computing of all standard features of drug-likeness (Daina et al., 2019). All targets of each ligand were presented in Excel files, and selected common targets of all ligands were submitted for validation using *in silico* molecular docking (*vide infra*).

##### 2.3.1.4 Kinase target screening

Protein kinases are ubiquitous regulators of cell physiology, and their pathological activity switches cells from normal to abnormal state and disease phenotype. Accordingly, kinases are therapeutic targets, and more than ten kinase inhibitors have been entered into clinical practice in the last 4 years. Selectivity would be a core concept in the discovery of novel kinase inhibitors. Thus, to find safe and potent NCEs, their interactions with kinases would be initial steps in drug development. In essence, KinScreen (https://www.way2drug.com/KinScreen/) was aimed to optimize this process. The key features of KinScreen include the prediction of kinase targets with PASS software, untangling molecular mechanism of action of NCEs, the visualization of results on the kinome tree, and the search for analogous compounds across the ChEMBL database to find experimental data on them that were ignored in this study.

##### 2.3.1.5 Molecular docking validation

The structures of synthesized ligands were converted into.pdb format using Open Babel (O’Boyle et al., 2011). The X-ray crystal structure of targets was retrieved from Protein Data Bank (PDB; http://www.RCSB.org), edited, optimized, and trimmed using Molegro Virtual Docker machine (Thomsen and Christensen, 2006) and Chimera 1.8.1 (http://www.rbvi.ucsf.edu/chimera). The *in silico* molecular docking was performed using PyRx software version 0.8 and the results were represented as binding affinity (BA; kcal/mol) values (Dallakyan and Olson, 2015).

#### 2.3.2 Bioinformatics

The UniProt accession identification of target kinases was converted to gene symbols for humans using the SynGO gene set analysis tool (Koopmans et al., 2019), and pooled together, and submitted to GeneMANIA to construct target kinase network. GeneMANIA is a handy web interface for acquiring gene ontology, scrutinizing gene lists, and highlighting genes for functional assays (Warde-Farley et al., 2010). After choosing *Homo sapiens* from the list of optional organisms, the genes of interest in the previous step were entered into the search bar and the results were collated and high-scored genes were culled for further discussion. Moreover, the protein-protein network was also constructed in STRING *ver*. 12 launched at https://string-db.org, and submitted to Cytoscape *ver*. 3.10.2 for network analysis using a novel Cytoscape plugin *cytoHubba* and visualization (Shannon et al., 2003).

## 3 Results and discussion

### 3.1 Chemical synthesis and validation

The physicochemical properties of synthesized ligands have been shown in (Supplementary Table 1).

#### 3.1.1 Characterization of)9H-xanthen-yl)hydrazine (L1)

The compound (L1) was synthesized by the reaction of xanthydrol with hydrazine hydrate. This compound was identified by FTIR spectroscopy by appearing (N-H, N-H2) stretching vibration at 3,200 and 3,500 cm^−1^ in compound (L1) and also the disappearance of the (O-H) bond at 3,345 cm^−1^ in xanthydrol (Supplementary Figure 1).

#### 3.1.2 Synthesis of potassium 2-)9H-xanthen-yl) hydrazinecarbodithoate (L2)

The compound (L2) was synthesized by the reaction of compound (L1) with carbon disulfide. This compound was identified by FTIR spectroscopy by the disappearance of the (N-H2) bond at 3,200–3,400 cm^−1^ in the compound and appearing of (C=S) stretching vibration at 1,270 cm^−1^ for compound (L2) (Supplementary Figure 2).

#### 3.1.3 Characterization of (E)-2-(2-(9H-xanthen-9-yl)hydrazono)-1,3-dithiolan-4-one (L3)

**FIGURE 1:**
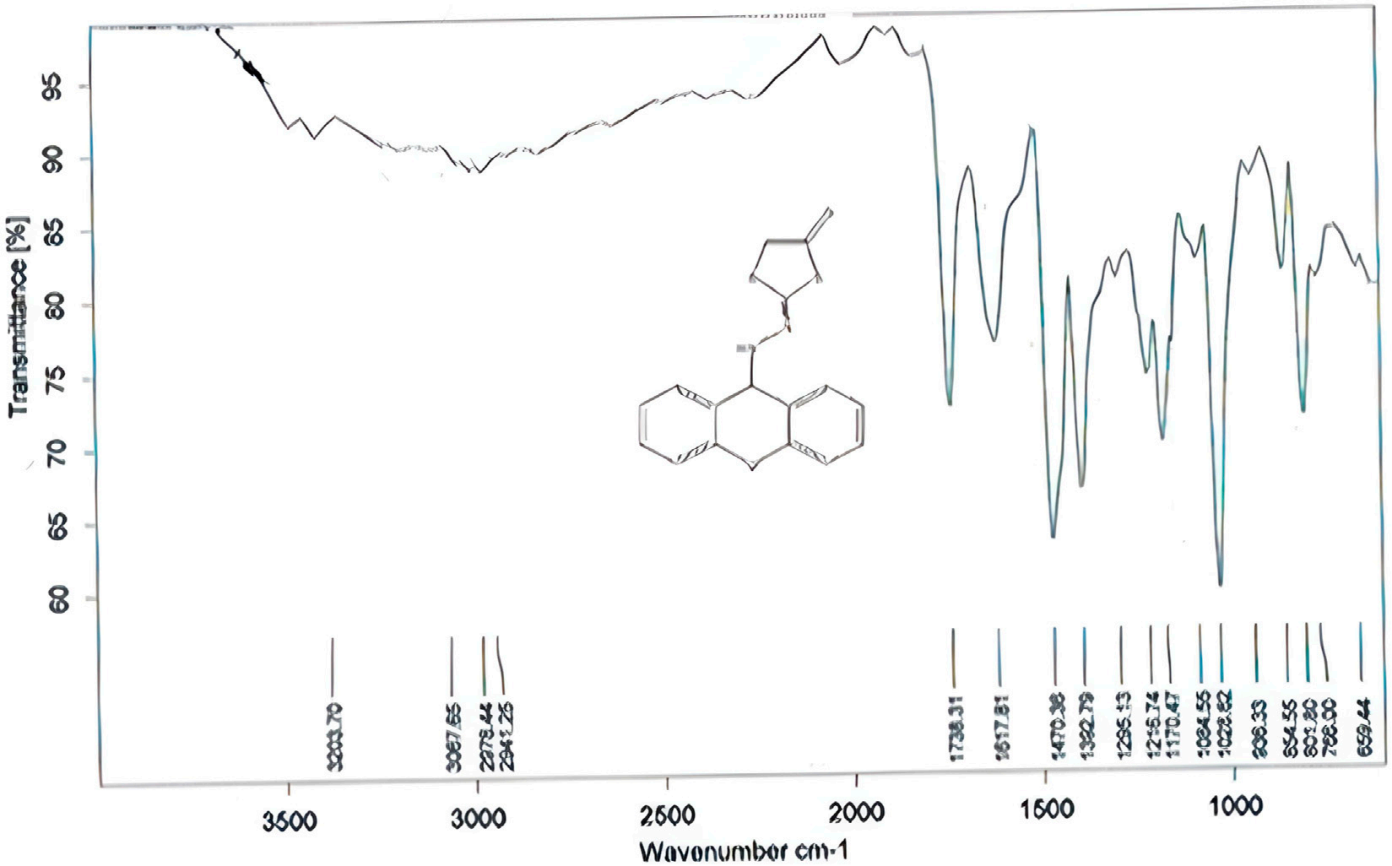
FTIR spectrum of compound (L3).

Compound (L3) was synthesized by the reaction of L2 with ethyl 2-chloroacetate. This compound was identified by FTIR spectroscopy by appearing (C=O) stretching vibration at 1,738 cm^−1^ and (N=C) at 1,671 cm^−1^ compound (L3) and also the disappearing the (C=S) bond at 1,207 cm^−1^ in compound (L2) (Figure 1).

#### 3.1.4 Characterization of 2-(9H-xanthe-9-yl)hydrazinecarbothioamide (L4)

The compound (L4) was synthesized by the reaction of xanthydrol with hydrazinecarbothioamide. These compounds were identified by FTIR spectroscopy by appearing (N-H, NH2) stretching vibration at 3,200 and 3,500 cm^−1^ for compounds (L4), respectively, and also the disappearing the (O-H) bond at 3,200–3,400 cm^−1^ in xanthydrol and appearance of (C=S) stretching vibration at 1,200 cm^−1^ for compound (L4) (Supplementary Figure 3). The mechanism of the reaction was shown (Supplementary Scheme 1).

#### 3.1.5 Characterization of 2-(2-(9H-xanthen-9-yl)hydrazinyl)thiazol-5(4H) one (L5)

**FIGURE 2:**
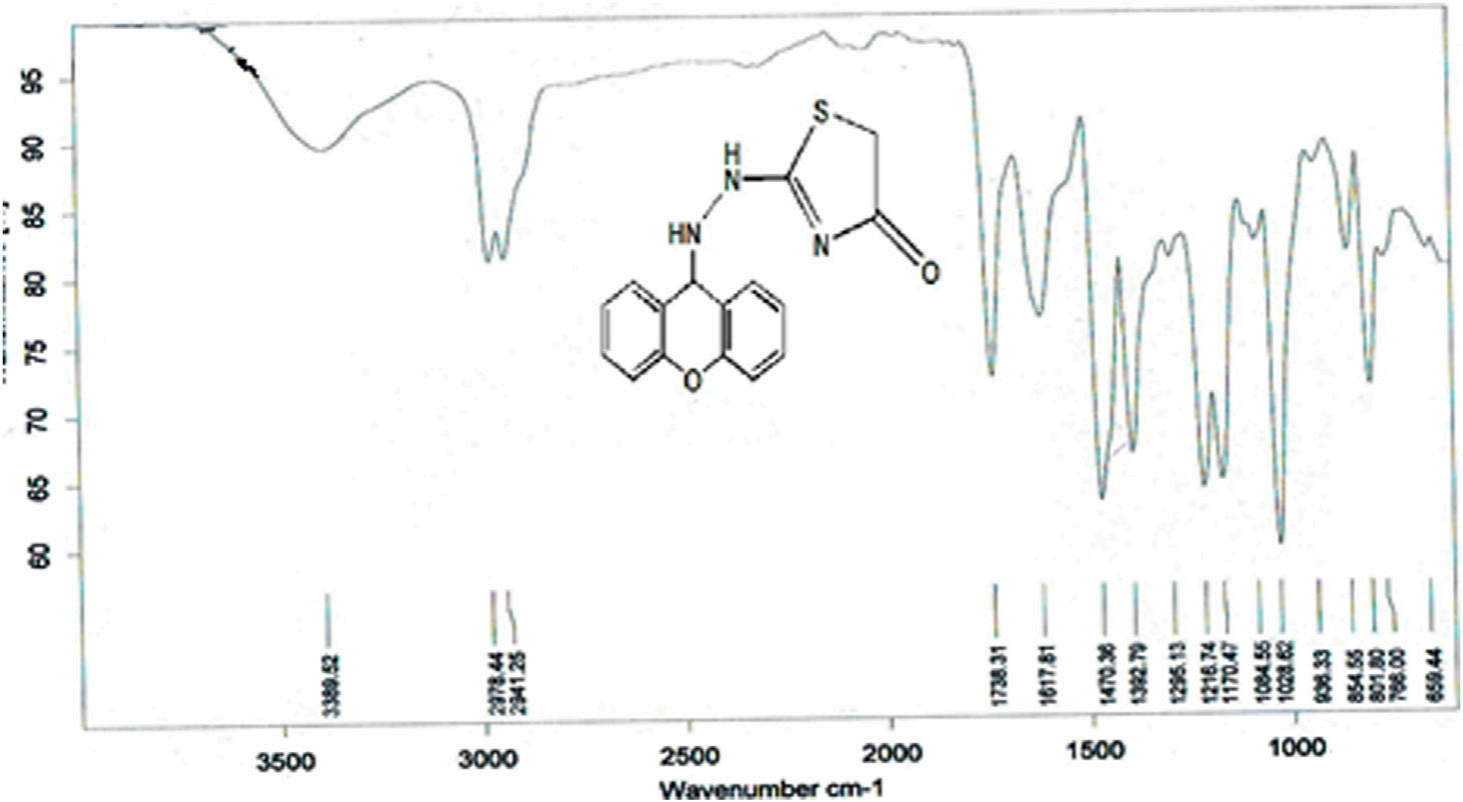
FTIR spectrum of compound (L5).

**FIGURE 3:**
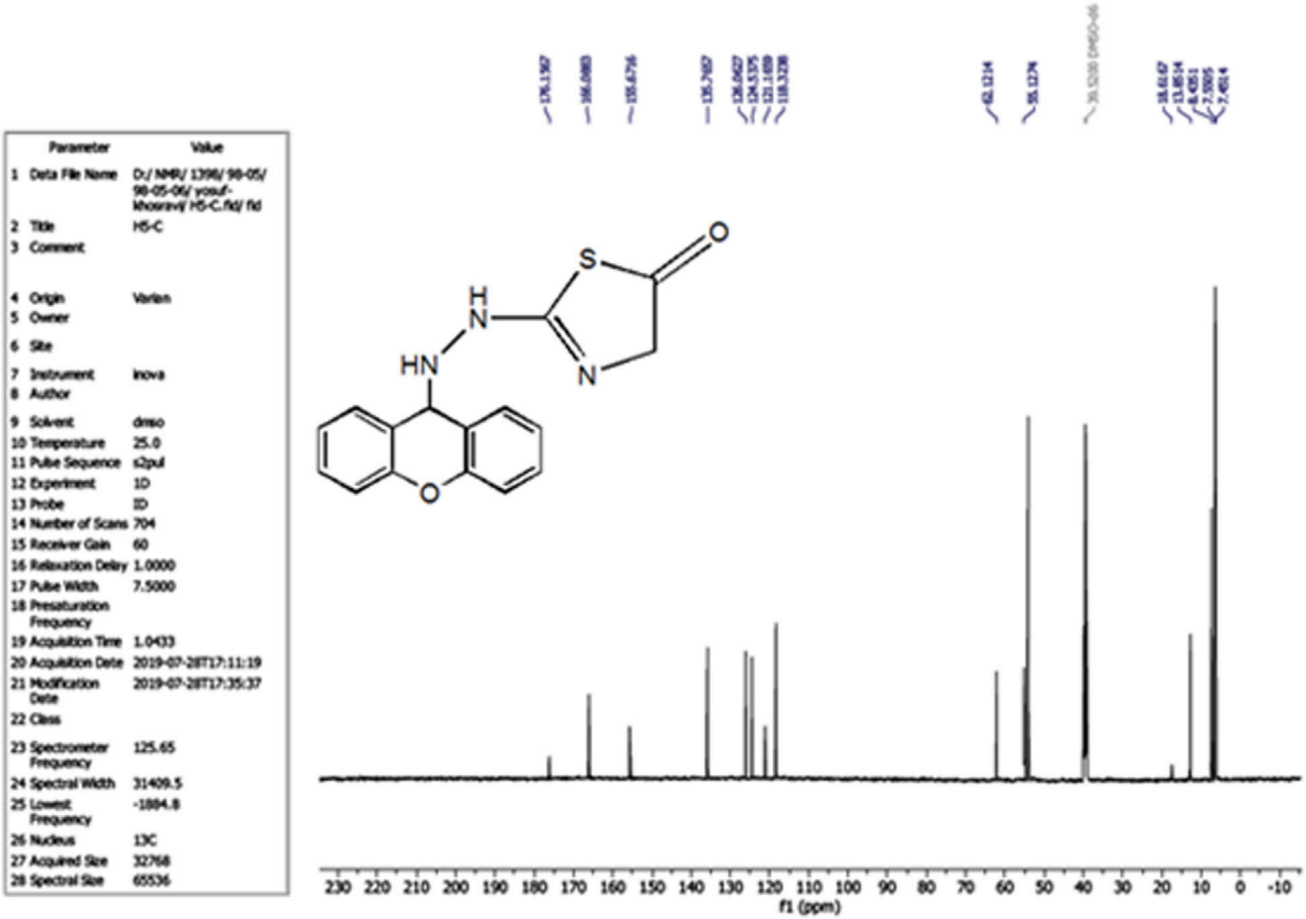
^13^C-NMR Spectrum of compound (L5).

**FIGURE 4:**
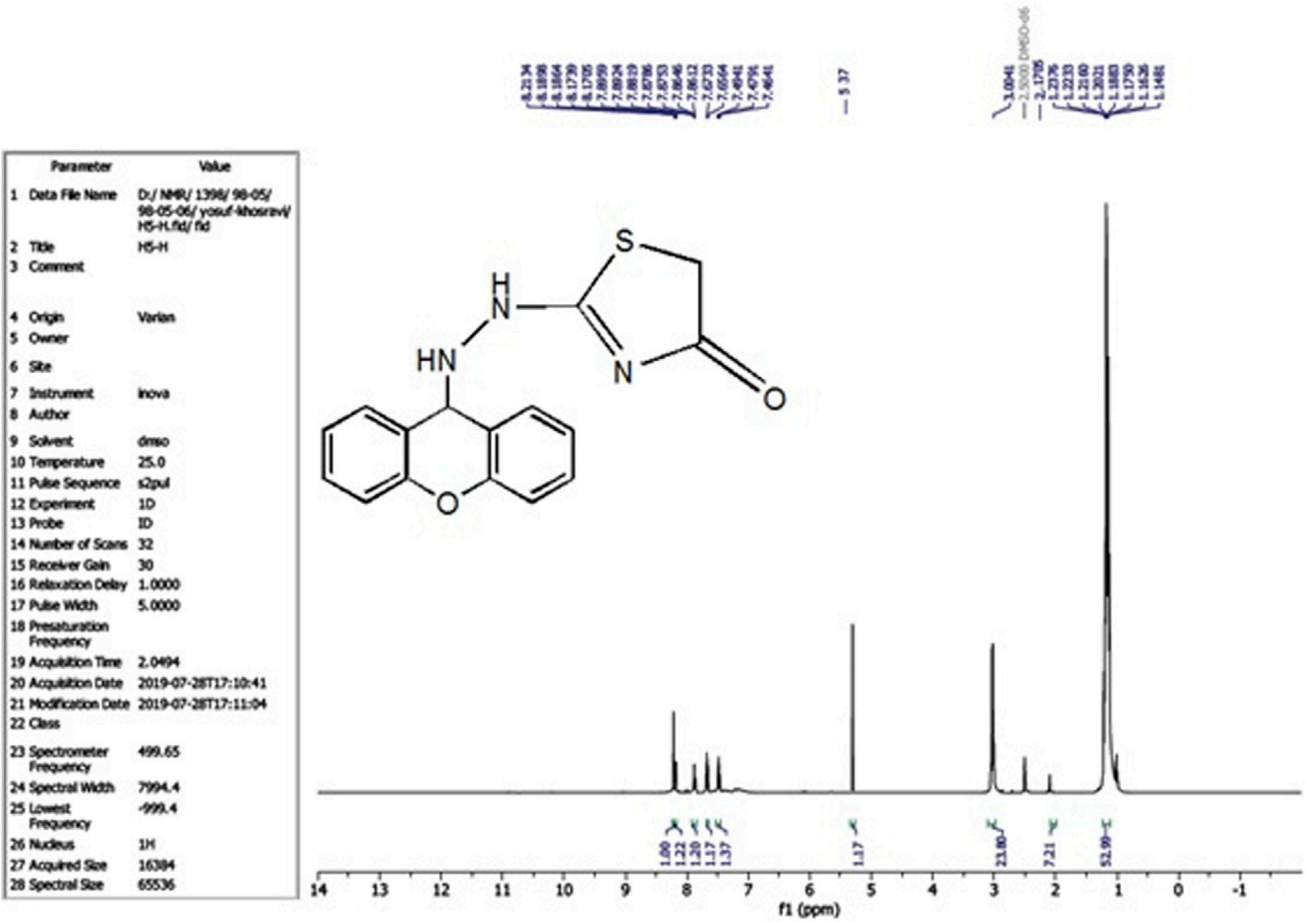
^1^H-NMR Spectrum of compound (L5).

**SCHEME 1:**
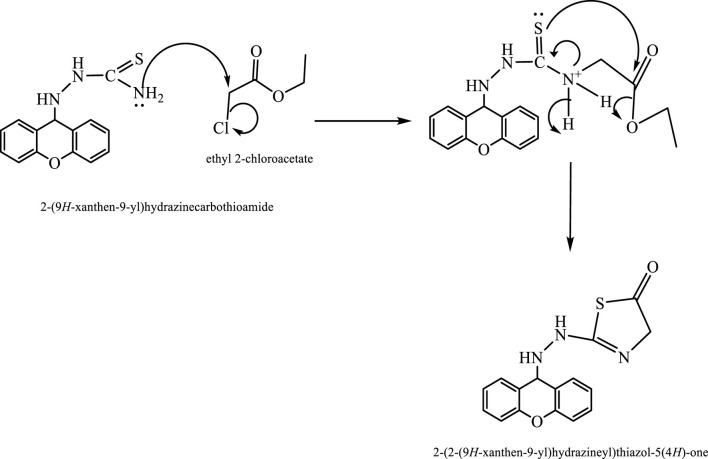
Mechanism of synthesis of compound (L5).

The compound (L5) was synthesized by the reaction of (L4) with ethyl 2-chloroacetate. This compound was identified by FTIR spectroscopy by appearing (C=O) stretching vibration at 1,731 cm^−1^ and disappearing of the (N-H2) bond at 3,200–3,400 cm^−1^ in compound (L4) and disappearing the (C=S) stretching vibration at 1,200 cm^−1^ for compound (L4), and appearing of (C=N) stretching vibration at 1,617 cm^−1^ for compound (L5) (Figure 2). ^13^C-NMR appearing (55) (C-N) 9H-xanthene (62) (=N-C)thiazol (130–140) Aromatic (166) (C=N)thiazol (176) (C=O)thiazol (155) (=C-O)9H-xanthene (Figure 3). The compound (L5) was also determined by the appearance of ^1^H-NMR from (NH) amine (1H) is about (2.1) while (2H) (=N-CH2) thiazol is at (3) and (CH-NH)9H-xanthene (1H) is at (5.3) while aromatic is at (7–8) (Figure 4). The reaction mechanism is depicted below (Scheme 1). The L5 (C16H13N3O2S; Figure 4) has been composed of C (59.49%–61.72%), H (4.21%–5.23%), N (13.50%–15.12%), and S (10.30%–10.71%).

#### 3.1.6 Characterization of 1-(9H-xanthen-9-yl)thiourea (L6)

The compound (L6) was synthesized by the reaction of xanthydrol with thiourea, and identified by FTIR spectroscopy by appearing (N-H, NH2) stretching vibration at 3,200 and 3,500 cm^−1^ for compound (L6) respectively, and also the disappearing the (O-H) bond at 3,200–3,400 cm^−1^ in xanthydrol, and appearing (C=S) stretching vibration at 1,209 cm^−1^ for compound (L6) (Supplementary Figure 4).

#### 3.1.7 Characterization of 2-(9H-xanthen-9-ylamino)thiazol-5(4H)-one (L7)

**FIGURE 5:**
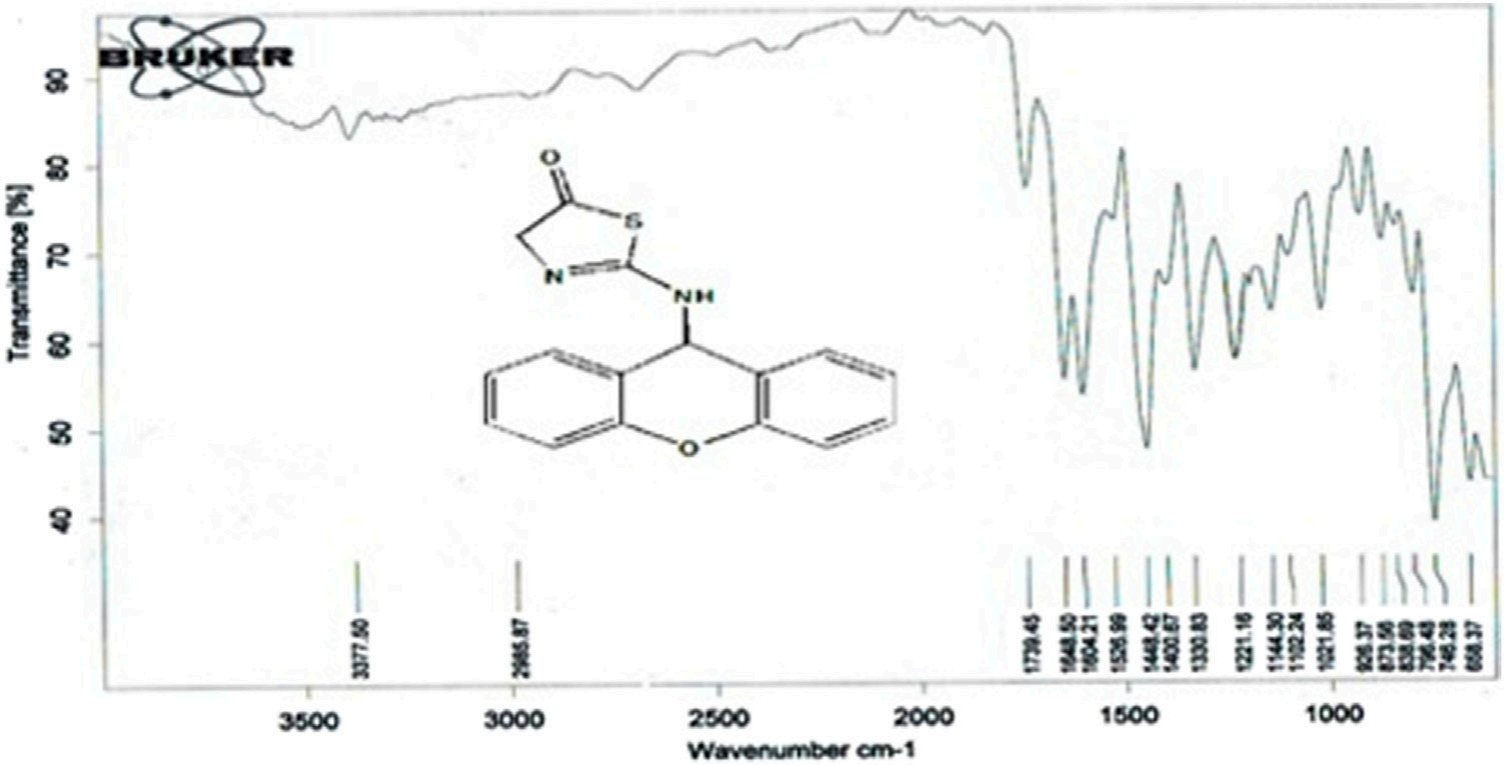
FTIR spectrum of compound (L7).

Compound (L7) was synthesized by the reaction of (L6) with ethyl 2-chloroacetate. This compound was identified by FTIR spectroscopy by appearing (C=O) stretching vibration at 1,739 cm^−1^ in compound (L7) and also the disappearing the (N-H2) bond at 3,200–3,400 cm^−1^ in compound (L6), and appearing of (C=N) stretching vibration at 1,648 cm^−1^ for compound (L7) (Figure 5).

#### 3.1.8 Characterization of 9-(prop-2-ynyloxy)-9H-xanthene (L8)

The compound (L8) was synthesized by the reaction of xanthydrol with propargyl bromide. This compound was identified by FTIR spectroscopy by appearing (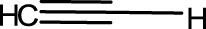) stretching vibration at 2,123 cm^−1^ for compounds (L8) and also the disappearing the (O-H) bond at 3,200–3,400 cm^−1^ in xanthydrol and appearing (C-H) vibration for compound (L8) (Supplementary Figure 5).

#### 3.1.9 Characterization of 4-((9H-xanthen-9-yloxy)methyl)-1-(4-nitrophenyl)-1H-1,2,3-triazole (L9)

**FIGURE 6:**
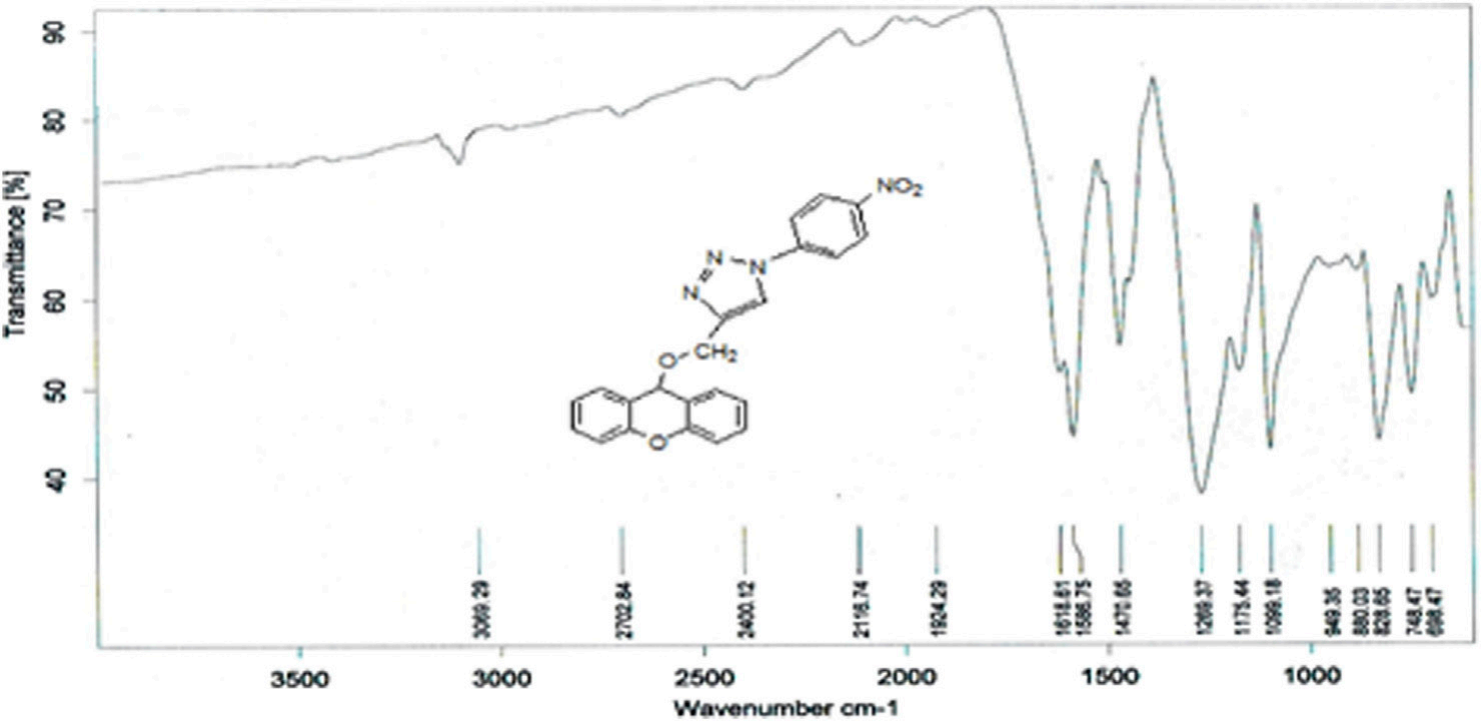
FTIR spectrum of compound (L9).

The compound (L9) was synthesized by the reaction of (L8) with 1-azido-4-nitrobenzene. This compound was identified by FTIR spectroscopy by appearing (NH) stretching vibration at 3,362 and 3,474 cm^−1^ for compound (L9), and also the disappearing the (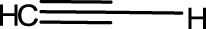) bond at 2,123 cm^−1^ in compound (L8) and disappearance of (C-H) vibration for compound (L8; Figure 6).

### 3.2 ADMET scan

**FIGURE 7:**
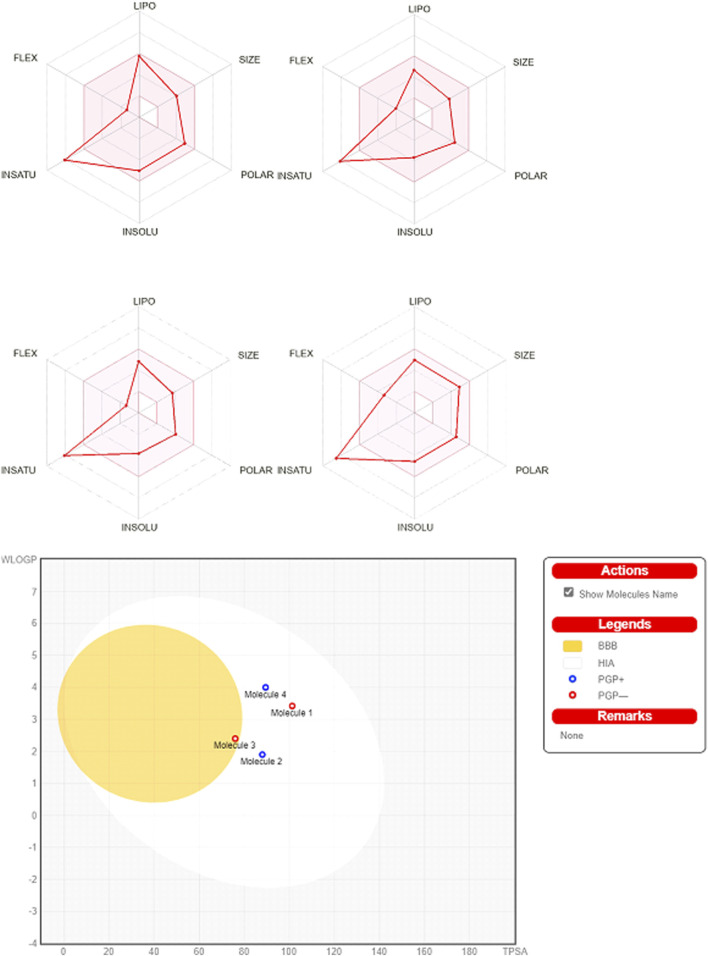
Bioavailability radars and BOILED-Egg scan. The pink area symbolizes the optimal range for physicochemical properties (size: MW (molecular weight; 150–500 g/mol, lipophilicity: XLOGP3 −0.7-+5.0, polarity: TPSA (topological polar surface area; 20–130Å^2^, solubility: log S ≤ 6, saturation: fraction of carbons in the Csp^3^ hybridization ≥0.25, and flexibility: ≤9 rotatable bonds. Note: upper left; 2-[2-(9H-xanthen-9-yl)hydrazinyl]-1,3-dithiolan-4-one (L3); upper right; 2-[2-(9H-xanthen-9-yl)hydrazinyl]-1,3-thiazol-5(4H)-one (L5); lower left; 2-(9H-xanthen-9-ylamino)-1,3-thiazol-5(4H)-one (L7), and lower right; hydroxy (oxo)(4-{4-[(9H-xanthen-9-yloxy)methyl]-1H-1,2,3-triazol-1-yl}phenyl)ammonium (L9). Human intestinal absorption (HIA), blood-brain barrier (BBB) penetration, lipophilicity (WLOGP), and polarity (TPSA) have been computed. The mutually exclusive white and region (yolk) region depict the highest probability of being absorbed by gut and being permeated to the brain, respectively.

Ligands obeyed the Lipinski (Pfizer) filter for drug-likeness without positive alerts for considering pan-assay interference compounds (PAINS; Supplementary Figure 7; Supplementary Table 1). PAINS react promiscuously to various targets because they possess disruptive functional groups that have been traced by SwissADME (Baell and Holloway, 2010; Daina et al., 2017). In this line, MWs of ligands were less than 500 g/mol and they can be absorbed from the gastrointestinal tract. MLOGP of L9 (3.2) was increased in comparison to other ligands (Table 1), while other ligands had identical MLOGP of 1.9 which reflects lesser lipophilicity of all ligands. The bioavailability scores of all ligands were the same. Among all ligands, L7 showed blood-brain barrier (BBB) permeation. The topical polar surface areas (TPSAs) of all ligands were less than 140 Å^2^ which obeys the RO5. The number of rotatable bonds, single bonds that can freely rotate around their axis, was higher in L9 as compared to those of other ligands. The number of rotatable bonds in a molecule affects its efficacy, pharmacokinetic properties, conformational flexibility, interaction with target proteins, and ADMET properties. Based on the higher number of rotatable bonds in L9, this ligand may be too flexible to bind tightly to its target. The flexibility of L9 was higher than those of other ligands which showed similar flexibility. In this regard, the number of rotatable bonds should not surpass nine rotatable bonds. The role of flexibility as a filter for ADMET properties besides TPSA, and hydrogen bond count are the key determinants of bioavailability (Veber et al., 2002). The number of rotatable bonds in the Csp^3^ configuration dictates the flexibility of a molecule (Caron et al., 2020). Although this configuration was decreased in L9 as compared to those of other ligands, it was more flexible. Among proper physicochemical properties of ligands for oral bioavailability (Figure 7), only saturation (fraction Csp^3^) was lesser than the cutoff value of 0.22. All ligands had fraction Csp3 values of 0.12 except L9 which had 0.09 (Supplementary Material S4). In this study, all synthesized ligands possessed a SP^3^ hybridization fraction lesser than 0.25. The percentage of SP^3^ fraction in a molecule is a main descriptor for the prediction of the three-dimensionality and intricacy of molecular structures. Computationally, the SP^3^ fraction is used to evaluate the “drug-likeness” or “lead-likeness” of NCEs, and molecules with a higher SP^3^ fraction often display better pharmacological profiles. These molecules are expected to adopt diverse spatial conformations, allowing more specific interactions with the intricate shapes of biological targets. In summary, synthesized ligands were accepted as lead-like compounds and they should be submitted to QSAR screening for their biological activities to optimize their pharmaceutical applications.

**TABLE 1:** Pharmacological and toxicological features of synthesized xanthone derivatives.

Ligand	MW g/mol	MLOGP	Hdon	Hacc	BS	BBB	TPSA Å^2^	RBN	Lipinski #violations	PAINS #alert
L3	328	1.99	1	3	0.55	NO	101.29	2	0	0
L5	311	1.97	2	4	0.55	No	88.02	3	0	0
L7	296	1.99	1	3	0.55	Yes	75.99	2	0	0
L9	401	3.26	1	6	0.55	No	89.48	5	0	0

Note: MW, molecular weight (kDa); MLOGP, Moriguchi Log *P*_*o/w*_; Hdon, the number of H-bond donors; Hacc, the number of H-bond acceptors; BS, oral bioavailability score; Caco-2, permeability; BBB, blood-brain barrier; TPSA, topographical polar surface area; RBN, the number of rotatable bonds: HL, half-life; PAINS, Pan-assay interference compounds. 2-[2-(9H-xanthen-9-yl)hydrazinyl]-1,3-dithiolan-4-one (L3; SMILE: O=C1CS\C(S1)=N/NC1C2=CC=CC=C2OC2=C1C=CC=C2), 2-[2-(9H-xanthen-9-yl)hydrazinyl]-1,3-thiazol-5(4H)-one (L5 SMILE: O=C1CN=C(NNC2C3=CC=CC=C3OC3=C2C=CC=C3)S1), 2-(9H-xanthen-9-ylamino)-1,3-thiazol-5(4H)-one (L7 SMILE: O=C1CN=C(NC2C3=CC=CC=C3OC3=C2C=CC=C3)S1), and lower right; hydroxy(oxo)(4-{4-[(9H-xanthen-9-yloxy)methyl]-1H-1,2,3-triazol-1-yl}phenyl)ammonium (L9 SMILE: ON(=O)C1=CC=C(C=C1)N1C=C(COC2C3=CC=CC=C3OC3=CC=CC=C23)N=N1).

All ligands had high human intestinal absorption (HIA), while L7 just displayed BBB permeation (Supplementary Material S4). L5 and L9 were substrates of P-glycoprotein (P-gp) as a subset of ATP-binding cassette (ABC) transporters that can efflux drugs of cells to shorten the duration of their pharmacological effects (Amezian and Van Leeuwen, 2024). Moreover, all synthesized ligands except L9 were cytochrome P450 (CYP450) inhibitors. CYP450 enzymes facilitate the phase I of xenobiotics metabolism to make them more soluble for excretion (Esteves et al., 2021). All NCEs should be computationally tested for categorizing as inhibitors or substrates of CYP450 enzymes to forecast their drug-drug interactions and ADRs (Daina et al., 2017). In this continuum, L3, L5, and L7 contain heterocyclic rings with complex substituents, while L9 contains functional groups associated with enhanced toxicity. All ligands were categorized as Class III of toxicity according to the Cramer decision tree (Cramer et al., 1976). In this line, the triazole ring is known as one of the main pharmacophores of the nitrogen-containing heterocycles (Guo et al., 2021), and it can enhance the toxicological activities of other molecules. Therefore, the synthesized ligands should be checked through a battery of bioassays for consideration as lead-like, drug, or poison.

### 3.3 Target fishing and validation

*Target fishing* is a bottleneck in the laborious and expensive pipeline of experimental pharmacology for dissecting the interaction of a bioactive compound with its druggable target proteins. In this continuum, *in silico* target fishing employs machine learning algorithms and chemi- and bio-informatic tools for the prediction of the targets of NCEs. Here, we used the SwissTargetPrediction to predict chemical-protein interactions (Supplementary Material S5). Four common targets of all ligands were extracted from target sets of each ligand and submitted to *in silico* molecular docking validation (Supplementary Material S6). Four common targets were three kinases including vascular endothelial growth factor receptor 2 (CHEMBL279; PDB ID: 1YWN), tyrosine-protein kinase (CHEMBL2148; PDB ID: 3LXL), and epidermal growth factor receptor (erbB1; CHEMBL203; PDB ID: 5UG9), and sodium channel protein type IX alpha subunit (CHEMBL4296; PDB ID: 5EKO) which belongs to the family of voltage-gated ion channels. In this context, L3 predominantly docked with family erbB1, however it interacted reliably with other kinases like MAP kinase ERK1 and protein kinase C mu (Table 2). In this continuum, L5 docked firmly with erbB1 and tyrosine-protein kinase, and it interacted with MAP kinase ERK1. L7 docked with tyrosine-protein kinase more reliably than other ligands, and it was the weakest binder in this study. Interestingly, L9 showed the strongest BAs with all common targets, while it docked tyrosine-protein kinase with the highest BA of −11.1 kcal/mol (Supplementary Material S6). In summary, these synthesized ligands docked reliably with tyrosine-protein kinase and erbB1 (Supplementary Material S6).

**TABLE 2:** *In silico* molecular docking [binding affinity (lower bound, upper bound)] of xanthone derivatives with top-list predicted targets.

Ligand	Top-list target (PDB)					
	1YWN	3LXL	5EK0	5UG9	3W8Q	4U7Z
L3	−7.8 (7.926; 4.45)	−8.8 (4.122; 1.989)	−8.1 (5.771; 3.003)	−9.0 (3.675; 0.069)	−8.7 (3.675; 0.116	−8.5 (3.676; 0.122)
L5	−8.2 (3.675; 0.074)	−9.1 (3.676; 0.093)	−7.0 (37.57; 34.943)	−9.0 (3.675; 0.062)	−9.5 (3.676; 0.1)	−8.9 (3.676; 0.104
L7	−7.9 (3.761; 0.008)	−8.5 (3.762; 0.065)	−6.9 (3.761; 0.027)	−8.3 (3.762; 0.049)	−8.5 (3.762; 0.017)	−8.3 (3.762; 0.016)
L9	−10.1 (3.261; 0.17)	−11.1 (3.259; 0.115)	−9.3 (2.786; 2.234)	−10.2 (3.265; 0.221)	−10.3 (3.92; 2.373)	−10.2 (3.258; 0.055)

Note**:** 2-[2-(9H-xanthen-9-yl)hydrazinyl]-1,3-dithiolan-4-one (L3), 2-[2-(9H-xanthen-9-yl)hydrazinyl]-1,3-thiazol-5(4H)-one (L5), 2-(9H-xanthen-9-ylamino)-1,3-thiazol-5(4H)-one (L7), and hydroxy (oxo)(4-{4-[(9H-xanthen-9-yloxy)methyl]-1H-1,2,3-triazol-1-yl}phenyl)ammonium (L9).

### 3.4 Kinase target fishing

**FIGURE 8:**
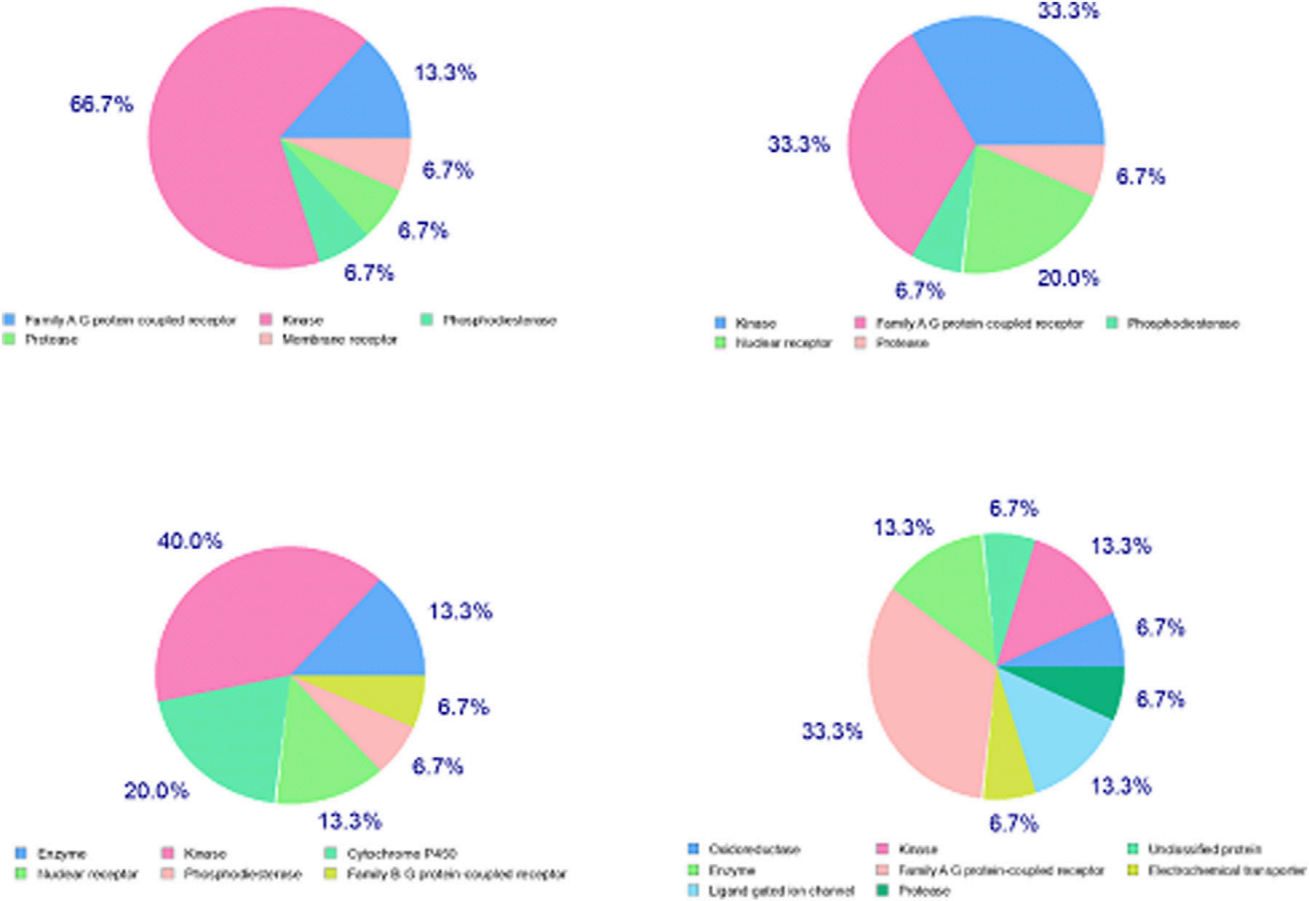
Distribution of targets in various classes for xanthones derivatives. Note: upper left; 2-[2-(9H-xanthen-9-yl)hydrazinyl]-1,3-dithiolan-4-one (L3); upper right; 2-[2-(9H-xanthen-9-yl)hydrazinyl]-1,3-thiazol-5(4H)-one (L5); lower left; 2-(9H-xanthen-9-ylamino)-1,3-thiazol-5(4H)-one (L7), and lower right; hydroxy (oxo)(4-{4-[(9H-xanthen-9-yloxy)methyl]-1H-1,2,3-triazol-1-yl}phenyl)ammonium (L9).

Except for L9, the major target family of synthesized ligands was the kinase family as predicted by SwissTarget (Figure 8). Moreover, KinScreen launched at https://www.way2drug.com/KinScreen/predicted the interaction of pharmacological substances with human kinome (see Supplementary Material S7). In essence, the main top 5 list targets and therapeutic classes of ligands have been presented (Supplementary Table 1). In this continuum, various serine/threonine-protein kinases were common kinase targets for synthesized ligands. Protein kinase C mu, MAP kinase ERK1, dual specificity protein kinase CLK3, and casein kinase I gamma 2 were highly accurate predicted targets for L3, L5, L7, and L9, respectively (Supplementary Table 1). Consistent with the results of KinScreen, SwissTarget also showed that the common targets for four synthesized ligands were three kinases and one target belongs to the voltage-gated ion channel (VIC) superfamily (Figure 8).

### 3.5 Kinase target network

All target kinases were harvested from KinScreen and submitted to GeneMANIA and STRING servers (*vide supra*). In this regard, three genes showed the highest weights in the kinase target network of synthesized ligands in the output of GeneMANIA (see Supplementary Table 1). In this line, haploid germ cell-specific nuclear protein kinase (Haspin) showed the maximum weight in the network constructed using the GeneMANIA server. This gene encodes a serine/threonine kinase that is known as a promising target against cancer (Liu et al., 2023). WEE2 is a kinase that is expressed in an array of cancers (https://www.proteinatlas.org/ENSG00000214102-WEE2/pathology). The PIM3 belongs to the Ser/Thr protein kinase family, and PIM subfamily is overexpressed in epithelial and hematological tumors and associated with MYC coexpression. It plays roles in signal transduction, cell proliferation and survival, and tumorigenesis like human hepatoblastoma cells (Marayati et al., 2022).

**FIGURE 9:**
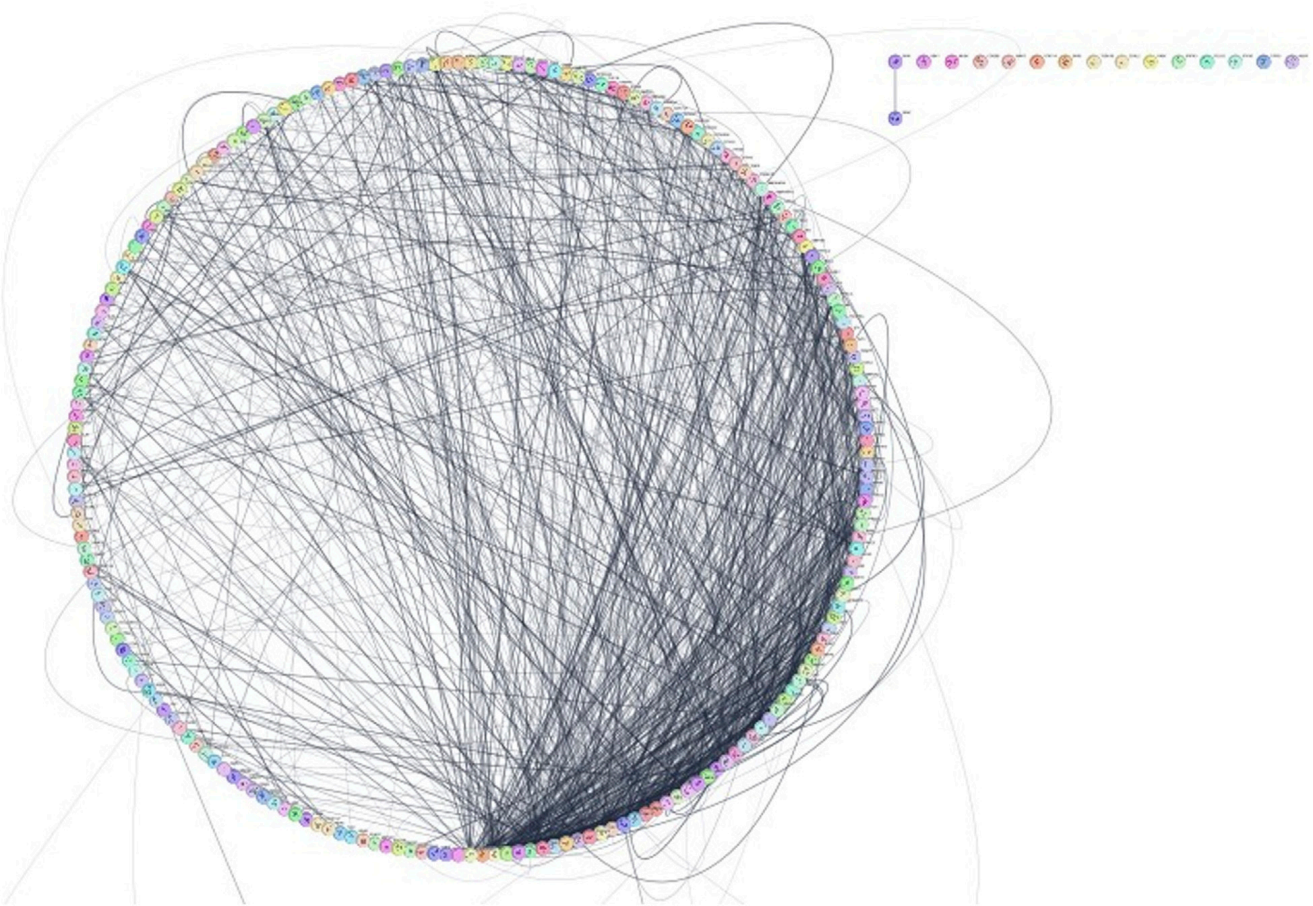
Visualization of the constructed network in Cytoscape results page.

**FIGURE 10:**
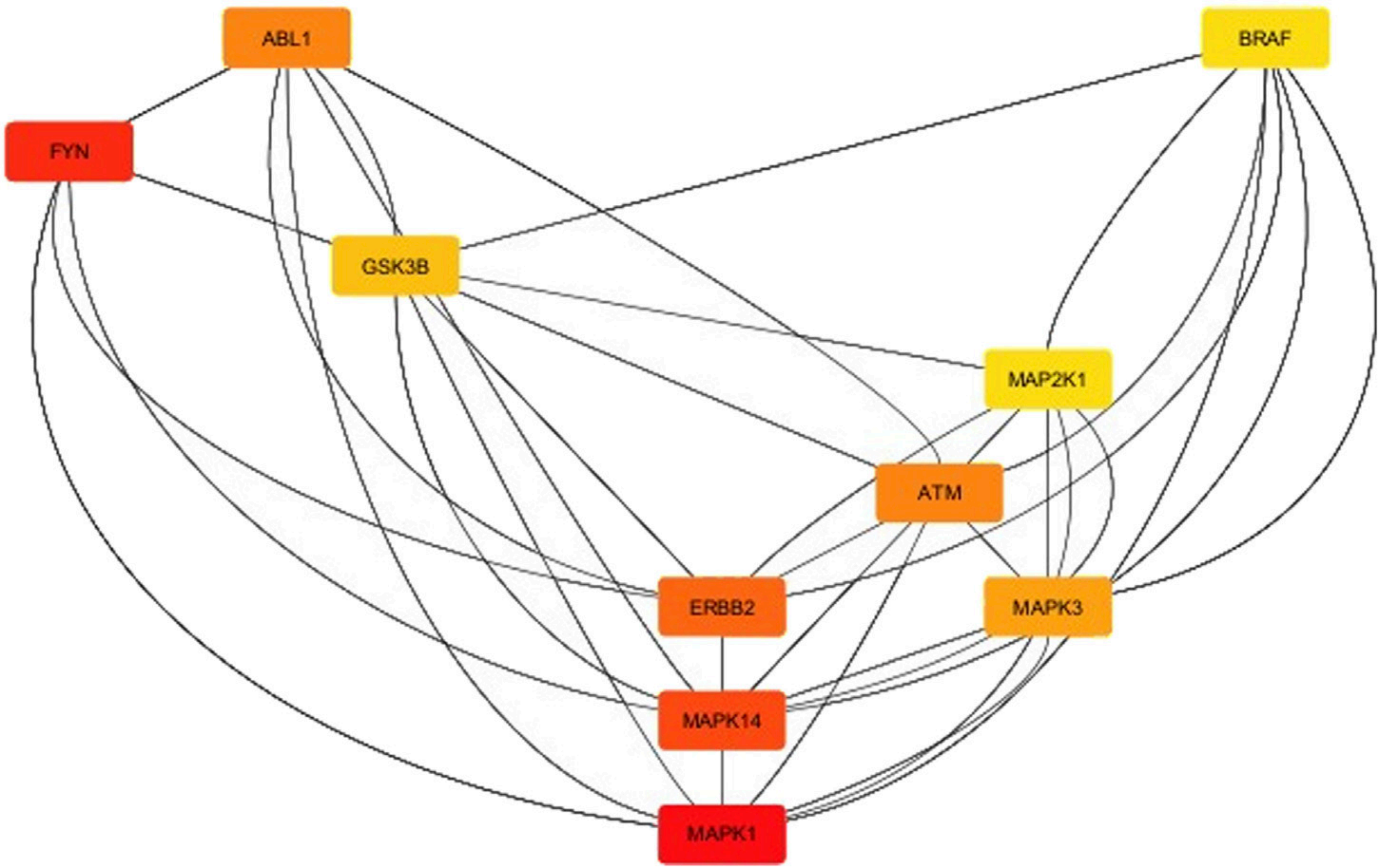
Top ten nodes ranked by degree using the CytoHubba app installed in Cytoscape.

The results of kinase network analysis were shown (Supplementary Material S9), and the degree sorted circle layout of the network was presented in Figure 9. Statistics of the network were summarized as follows: number of nodes: 222; number of edges: 1,344; average number of neighbors: 13,039; network diameter: 6; network radius: 3; characteristic path length: 2,601; clustering coefficient: 0.404; network density: 0.064; network heterogeneity: 0.893; network centralization: 0.231; connected components: 16; analysis time (sec): 0.152. In this line, the top-listed nodes with the highest degree (*n*) were MAPK1 (*n* = 60), FYN (*n* = 53), MAPK14 (*n* = 52), ERBB2 (*n* = 51), ABL1 (*n* = 47), ATM (*n* = 47), MAPK3 (*n* = 46), and GSK3B (*n* = 44) (see Supplementary Material S9). CytoHubba App was employed to search the hub node of the kinase target network (see Supplementary Material S10). Then, the top ten nodes ranked by degree were presented in Figure 10. Mitogen-activated protein kinase 1 (MAPK1) was the main *hub gene* among all target kinases for synthesized ligands which were submitted to molecular docking for further validation (Supplementary Table 2). We intentionally used two PDB IDs of MAPK1 to check the accuracy of BAs of synthesized ligands with a hub target that was determined through PPI network analysis. In essence, similar binding modes of synthesized ligands with MAPK1 PDBs were seen. In this case, L9 presented the best BA with MAPK1 (Table 2). MAPK1 is a serine/threonine kinase in the MAP kinase signal transduction pathway that is involved in the pathogenesis of many diseases and various types of cancers (Kim and Choi, 2010).

### 3.6 Antitarget and ADRs screening

Computation of the interaction of NCEs with *antitargets* is a crucial step to disclosing ADRs in the pipeline of drug discovery and development and clinical practice. In this regard, previous experimental data that are presented as K_i,_ K_act_, and IC_50_ values can be categorized and used as models to create (quantitative) structure and activity relationship [(Q)SAR] (Lagunin et al., 2018). In this study, we used GUSAR software launched at http://www.pharmaexpert.ru/GUSAR/antitargets.html, finds antitargets of hit of interest among about 4,000 chemicals interacting with 18 antitarget proteins (13 receptors, 2 enzymes and 3 transporters; see Supplementary Material S8). Based on GUSAR, rational accuracy (Q^2^_model_ > 0.6 and R^2^_test_ > 0.6) has been considered for computational prediction of antitargets of NCEs (Zakharov et al., 2012). All ligands showed altering activity of carbonic anhydrase II (see Supplementary Table 2) that may be the cause of aplastic anemia, anxiety, bone marrow suppression, chronic fatigue syndrome, alopecia, anaphylaxis, depression, and renal tubular acidosis (Rang et al., 1999). The interaction of synthesized ligands with 5-hydroxytryptamine 2C receptors as antagonists were also significant. In this case, the ADRs of antagonists to 5-hydroxytryptamine receptors are sickness, emesis, diarrhea, insomnia, and anxiety (Hoyer, 2020).

The specific potential of interacting with antitargets was also observed among our synthesized ligands. In this regard, trustful interaction of L3 was found with the 5-hydroxytryptamine 2A receptor and d3 dopamine receptor. Similarly, L5 also interacted with alpha1b adrenergic receptors. Adrenergic receptor antagonists may cause reflex tachycardia, orthostatic hypotension, nasal congestion, insomnia, and palpitation (Rang et al., 1999). L7 also interacted 5-hydroxytryptamine 2C receptor, while L9 interacted overtly with the kappa-type opioid receptor (see Supplementary Table 2). Antagonism to opioid receptors may cause emesis, sickness, respiratory depression, and sedation (Brunton et al., 2023). All synthesized ligands showed hepatotoxic potentials, while L7 presented cardiac toxicity (see Supplementary Table 3). Therefore, these ligands may possess some ADRs that must be considered in future experimental bioassays.

### 3.7 Antitumor potential

The cytotoxicity of NCEs against tumor cell lines is in the initial stages of development, repositioning, and drug research. However, the interaction of NCEs with normal cell lines gives impressive cues regarding their toxic potential. In this line, all synthesized ligands showed a low probability of activity against common normal cell lines that were used for snap-screening of cytotoxicity (see Supplementary Table 4). All ligands except L9, showed interaction with prostate epithelial cells. Embryonic lung fibroblast was also a target of all ligands except L7. In this context, L7 was less toxic for the normal cell line among ligands, and L3 showed a broad spectrum of toxicities against the normal cell lines.

Acute leukemic T-cells display one of the top predicted tumor cell lines for L5 (see Supplementary Table 5). All ligands except L9 showed weak interaction with glioblastoma and oligodendroglioma, and L9 showed a different pattern of tissue tumor targeting among ligands (see Supplementary Table 5). Allanxanthone C and macluraxanthone, two phyto-xanthones, exhibited proapoptotic and antiproliferative activities in leukemic cells from chronic lymphocytic leukemia and leukemia B cell lines (Loisel et al., 2010). Future investigations are welcomed to dissect the mechanism of possible antileukemic effects of synthesized xanthone derivatives that proposed in this study.

## 4 Conclusion

In conclusion, the total synthesis of nine novel xanthone derivatives was accomplished in a multi-step linear sequence and characterized. Finally, four compounds (L3, L5, L7, and L9) were selected for the computational assessment based on the Pfizer filter. Kinase network analysis showed that the top-listed nodes were MAPK1, FYN, MAPK14, ERBB2, ABL1, ATM, MAPK3, and GSK3B. MAPK1 was the main *hub gene* involved in the pathogenesis of many diseases and cancers. Interestingly, future investigations are needed to dissect the mechanism of possible antileukemic effects of synthesized xanthone derivatives that proposed in this study.

## Data Availability

The datasets presented in this study can be found in online repositories. The names of the repository/repositories and accession number(s) can be found in the article/Supplementary Material.
