# Corrigendum: Synthesis, characterization, and computational evaluation of some synthesized xanthone derivatives: focus on kinase target network and biomedical properties

**DOI:** 10.3389/fphar.2025.1583894

**Published:** 2025-04-23

**Authors:** Wisam Taher Muslim, Layth Jasim Mohammad, Munaf M. Naji, Isaac Karimi, Matheel D. Al-Sabti, Majid Jabir, Mazin A. A. Najm, Helgi B. Schiöth

**Affiliations:** ^1^ Department of Pharmaceutical Chemistry, College of Pharmacy, Kufa University, Najaf City, Iraq; ^2^ Department of Microbiology, College of Medicine, Babylon University, Hilla City, Iraq; ^3^ Clinical Laboratory Sciences, College of Pharmacy, Kufa University, Najaf City, Iraq; ^4^ Research Group of Bioengineering and Biotechnology, Laboratory for Computational Physiology, Department of Biology, Faculty of Science, Razi University, Kermanshah, Iran; ^5^ Department of Science, College of Science, Uruk University, Baghdad, Iraq; ^6^ Department of Applied Science, University of Technology, Baghdad, Iraq; ^7^ Department of Pharmacy, Mazaya University Collage, Nasiriyah, Iraq; ^8^ Department of Surgical Sciences, Functional Pharmacology and Neuroscience, Uppsala University, Uppsala, Sweden

**Keywords:** xanthone, total synthesis, ADMET, molecular docking, network analysis, target fishing

In the published article, there was an error in the affiliations.

The authors’ affiliations were incorrectly written as follows:

Wisam Taher Muslim^1^, Layth Jasim Mohammad^2^, Munaf M. Naji^3^, Isaac Karimi^4,5^*, Matheel D. Al-Sabti^6^, Majid Jabir^7^, Mazin A. A. Najm^8^ and Helgi B. Schiöth^5*^

^1^Department of Pharmaceutical Chemistry, College of Pharmacy, Kufa University, Najaf City, Iraq, ^2^Department of Microbiology, College of Medicine, Babylon University, Hilla City, Iraq, ^3^Clinical Laboratory Sciences, College of Pharmacy, Kufa University, Najaf City, Iraq, ^4^Research Group of Bioengineering and Biotechnology, Laboratory for Computational Physiology, Department of Biology, Faculty of Science, Razi University, Kermanshah, Iran, ^5^Department of Surgical Sciences, Functional Pharmacology and Neuroscience, Uppsala University, Uppsala, Sweden, ^6^Department of Science, College of Science, Uruk University, Baghdad, Iraq, ^7^Department of Applied Science, University of Technology, Baghdad, Iraq, ^8^Department of Pharmacy, Mazaya University Collage, Nasiriyah, Iraq.

The correct affiliations are:

“Wisam Taher Muslim^1^, Layth Jasim Mohammad^2^,Munaf M. Naji^3^, Isaac Karimi^4^, Matheel D. Al-Sabti^5^, Majid Jabir^6^, Mazin A. A. Najm^7^ and Helgi B. Schiöth^8^

^1^Department of Pharmaceutical Chemistry, College of Pharmacy, Kufa University, Najaf City, Iraq, ^2^Department of Microbiology, College of Medicine, Babylon University, Hilla City, Iraq, ^3^Clinical Laboratory Sciences, College of Pharmacy, Kufa University, Najaf City, Iraq, ^4^Research Group of Bioengineering and Biotechnology, Laboratory for Computational Physiology, Department of Biology, Faculty of Science, Razi University, Kermanshah, Iran, ^5^Department of Science, College of Science, Uruk University, Baghdad, Iraq, ^6^Department of Applied Science, University of Technology, Baghdad, Iraq, ^7^Department of Pharmacy, Mazaya University Collage, Nasiriyah, Iraq, ^8^Department of Surgical Sciences, Functional Pharmacology and Neuroscience, Uppsala University, Uppsala, Sweden”.

The authors apologize for this error and state that this does not change the scientific conclusions of the article in any way. The original article has been updated.

