# Supplementary material for: Synthesis, characterization, and computational evaluation of some synthesized xanthone derivatives: focus on kinase target network and biomedical properties
Source: Front Pharmacol. 2025 Jan 3;15:1511627. doi: 10.3389/fphar.2024.1511627 (PMC11738930; doi:10.3389/fphar.2024.1511627)
Supplement: Supplementary file 1 [file DataSheet1.zip › Supplementary file 2.DOCX]

**Synthesis, Characterization, and Computational Evaluation of Some Synthesized Xanthone Derivatives: Focus on Kinase Target Network and Biomedical Properties**

**Wisam Taher Muslim^1^, Layth Jasim Mohammad^2¥^, Munaf M. Naji^3^, Isaac Karimi^4,5^*^¥^, Matheel D. Al-Sabti^56^, Majid Jabir^7^, Mazin A. A. Najm^8^, Helgi B. Schiöth^5^*^¥^**

^1^Department of Pharmaceutical Chemistry, College of Pharmacy, Kufa University, Najaf City, Najaf Governorate, 540011, Iraq.

^2^ Department of Microbiology, College of Medicine, Babylon University, Hilla City, Babylon Governorate, 51002, Iraq.

^3^Clinical-Laboratory Sciences, College of Pharmacy, Kufa University, Najaf City, Najaf Governorate, 540011, Iraq.

^4*^Reseach Group of Bioengineering and Biotechnology, Laboratory for Computational Physiology; Department of Biology, Faculty of Science, Razi University 67149-67346, Kermanshah, Iran.

^5*^Department of Surgical Sciences, Functional Pharmacology and Neuroscience, Uppsala University, 751 24, Uppsala, Sweden.

^6^Department of Science, College of Science, Uruk University, Baghdad, Iraq.

^7^Department of Applied Science, University of Technology, Baghdad, Iraq.

^8^Department of Pharmacy, Mazaya University Collage, Nasiriyah, Thi-Qar, Iraq.

^¥^These authors contributed equally to this work

**Corresponding authors**: Helgi B. Schiöth, [helgi.schioth@uu.se](mailto:helgi.schioth@uu.se), Tel and Fax: 0046-18-4714160; Isaac Karimi; [isaac-karimi2000@yahoo.com](mailto:isaac-karimi2000@yahoo.com); [karimiisaac@razi.ac.ir](mailto:karimiisaac@razi.ac.ir). Tel & Fax: 0098-83-34274545.

**Table 1**. The physico-chemical properties of synthesized chemicals

| Chemical | Molecular formula | Molecular weight | Color | Melting point$\boldsymbol{℃}$ | Yield% | R.f. * | Solvent* mixture 2:6 ratio |
| --- | --- | --- | --- | --- | --- | --- | --- |
| L1 | C13H12N2O | 212.25 | White | 166-168 | 85 | 0.75 | Ben:EtOH |
| L2 | C14H11KS2N2O | 326.47 | Yellow | 111-113 | 79 | 0.58 | MtOH:EtOH |
| L3 | C16H12S2N2O2 | 330.42 | Nutty | 131-133 | 75 | 0.72 | MtOH:EtOH |
| L4 | C14H13SN3O | 404.46 | Yellow | 149-151 | 85 | 0.64 | Ben:EtOH |
| L5 | C16H13SN3O2 | 311.36 | Dark  brown | 134-136 | 80 | 0.49 | Ben:EtOH |
| L6 | C14H12SN2O | 256.32 | Brown | 147-149 | 85 | 0.60 | Ben:EtOH |
| L7 | C16H12SN2O2 | 256.32 | Nutty | 132-134 | 85 | 0.71 | Tol:EtOH |
| L8 | C15H12O2 | 224.25 | Nutty | 110-112 | 75 | 0.52 | Ben:EtOH |
| L9 | C22H16N4O4 | 400.38 | Brown | 157-159 | 85 | 0.52 | Ben:EtOH |

Note: * Rf.: retention factor; Ben: benzene; EtOH: ethanol; MtOH: methanol; Tol: toluene; Benz: benzene
