# Supplementary material for: Synthesis, characterization, and computational evaluation of some synthesized xanthone derivatives: focus on kinase target network and biomedical properties
Source: Front Pharmacol. 2025 Jan 3;15:1511627. doi: 10.3389/fphar.2024.1511627 (PMC11738930; doi:10.3389/fphar.2024.1511627)
Supplement: Supplementary file 1 [file DataSheet1.zip › Supplementary file 3.DOCX]

**Synthesis, Characterization, and Computational Evaluation of Some Synthesized Xanthone Derivatives: Focus on Kinase Target Network and Biomedical Properties**

**Wisam Taher Muslim^1^, Layth Jasim Mohammad^2¥^, Munaf M. Naji^3^, Isaac Karimi^4,5^*^¥^, Matheel D. Al-Sabti^56^, Majid Jabir^7^, Mazin A. A. Najm^8^, Helgi B. Schiöth^5^*^¥^**

^1^Department of Pharmaceutical Chemistry, College of Pharmacy, Kufa University, Najaf City, Najaf Governorate, 540011, Iraq.

^2^ Department of Microbiology, College of Medicine, Babylon University, Hilla City, Babylon Governorate, 51002, Iraq.

^3^Clinical-Laboratory Sciences, College of Pharmacy, Kufa University, Najaf City, Najaf Governorate, 540011, Iraq.

^4*^Reseach Group of Bioengineering and Biotechnology, Laboratory for Computational Physiology; Department of Biology, Faculty of Science, Razi University 67149-67346, Kermanshah, Iran.

^5*^Department of Surgical Sciences, Functional Pharmacology and Neuroscience, Uppsala University, 751 24, Uppsala, Sweden.

^6^Department of Science, College of Science, Uruk University, Baghdad, Iraq.

^7^Department of Applied Science, University of Technology, Baghdad, Iraq.

^8^Department of Pharmacy, Mazaya University Collage, Nasiriyah, Thi-Qar, Iraq.

^¥^These authors contributed equally to this work

**Corresponding authors**: Helgi B. Schiöth, [helgi.schioth@uu.se](mailto:helgi.schioth@uu.se), Tel and Fax: 0046-18-4714160; Isaac Karimi; [isaac-karimi2000@yahoo.com](mailto:isaac-karimi2000@yahoo.com); [karimiisaac@razi.ac.ir](mailto:karimiisaac@razi.ac.ir). Tel & Fax: 0098-83-34274545.


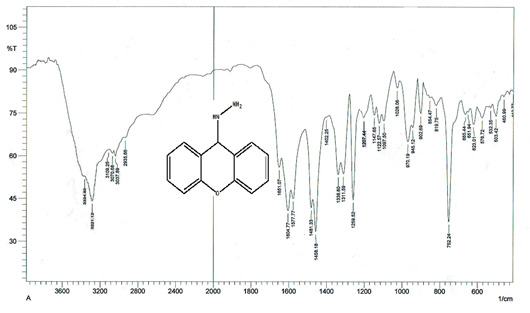


**Figure 1.** FTIR spectrum of compound L1

**
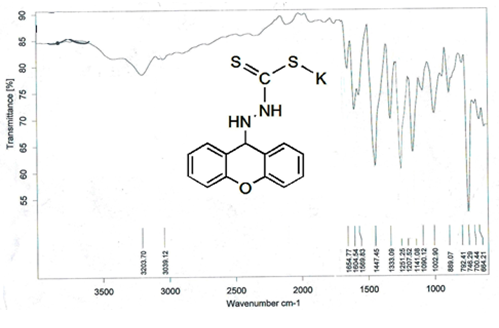
**

**Figure 2.** FTIR spectrum of compound L2

|  |
| --- |

**Scheme 1.** Mechanism of synthesis of compound L4


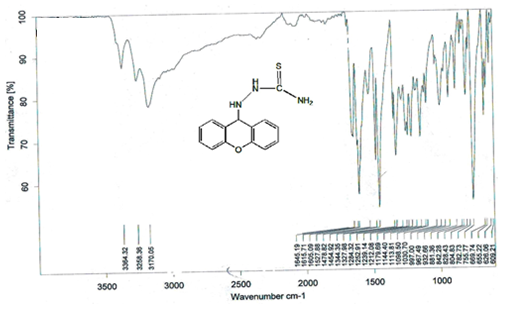


**Figure 3.** FTIR spectrum of compound L4

**
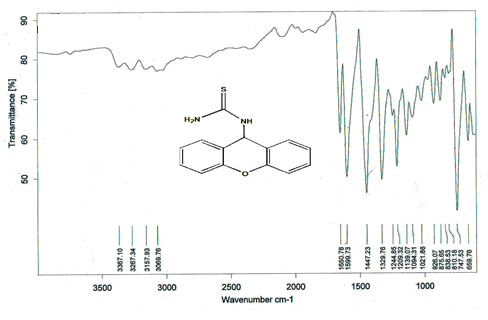
**

**Figure 4.** FTIR spectrum of compound L6

**
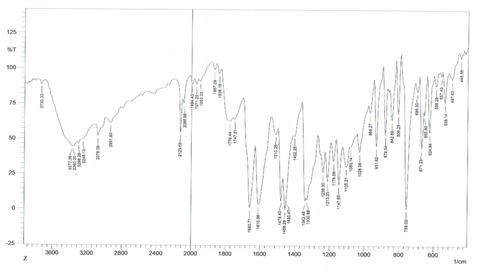
**

**Figure 5.** FTIR spectrum of Compound L8
