# Supplementary material for: Synthesis, characterization, and computational evaluation of some synthesized xanthone derivatives: focus on kinase target network and biomedical properties
Source: Front Pharmacol. 2025 Jan 3;15:1511627. doi: 10.3389/fphar.2024.1511627 (PMC11738930; doi:10.3389/fphar.2024.1511627)
Supplement: Supplementary file 1 [file DataSheet1.zip › Supplementary file 6.DOCX]

**Synthesis, Characterization, and Computational Evaluation of Some Synthesized Xanthone Derivatives: Focus on Kinase Target Network and Biomedical Properties**

**Wisam Taher Muslim^1^, Layth Jasim Mohammad^2¥^, Munaf M. Naji^3^, Isaac Karimi^4,5^*^¥^, Matheel D. Al-Sabti^56^, Majid Jabir^7^, Mazin A. A. Najm^8^, Helgi B. Schiöth^5^*^¥^**

^1^Department of Pharmaceutical Chemistry, College of Pharmacy, Kufa University, Najaf City, Najaf Governorate, 540011, Iraq.

^2^ Department of Microbiology, College of Medicine, Babylon University, Hilla City, Babylon Governorate, 51002, Iraq.

^3^Clinical-Laboratory Sciences, College of Pharmacy, Kufa University, Najaf City, Najaf Governorate, 540011, Iraq.

^4*^Reseach Group of Bioengineering and Biotechnology, Laboratory for Computational Physiology; Department of Biology, Faculty of Science, Razi University 67149-67346, Kermanshah, Iran.

^5*^Department of Surgical Sciences, Functional Pharmacology and Neuroscience, Uppsala University, 751 24, Uppsala, Sweden.

^6^Department of Science, College of Science, Uruk University, Baghdad, Iraq.

^7^Department of Applied Science, University of Technology, Baghdad, Iraq.

^8^Department of Pharmacy, Mazaya University Collage, Nasiriyah, Thi-Qar, Iraq.

^¥^These authors contributed equally to this work

**Corresponding authors**: Helgi B. Schiöth, [helgi.schioth@uu.se](mailto:helgi.schioth@uu.se), Tel and Fax: 0046-18-4714160; Isaac Karimi; [isaac-karimi2000@yahoo.com](mailto:isaac-karimi2000@yahoo.com); [karimiisaac@razi.ac.ir](mailto:karimiisaac@razi.ac.ir). Tel & Fax: 0098-83-34274545.

**Table 1.** *In silico* top-list predicted kinase targets of xanthones derivatives

| Compound | Name | UniProt ID | ChEMBL ID | Prediction accuracy (AUC, LOO CV) | Confidence |
| --- | --- | --- | --- | --- | --- |
| L3 | MAP kinase ERK1 | P27361 | CHEMBL3385 | 0.74 | 0.59 |
|  | Serine/threonine-protein kinase NEK6 | Q9HC98 | CHEMBL4309 | 0.7 | 0.57 |
|  | Serine/threonine-protein kinase 11 | Q15831 | CHEMBL5606 | 0.7 | 0.48 |
|  | Casein kinase I alpha | P48729 | CHEMBL2793 | 0.69 | 0.48 |
|  | Protein kinase C mu | Q15139 | CHEMBL3863 | 0.8 | 0.47 |
| L5 | Serine/threonine-protein kinase NEK6 | Q9HC98 | CHEMBL4309 | 0.7 | 0.63 |
|  | CaM kinase IV | Q16566 | CHEMBL2494 | 0.72 | 0.57 |
|  | Serine/threonine-protein kinase 11 | Q15831 | CHEMBL5606 | 0.7 | 0.56 |
|  | MAP kinase ERK1 | P27361 | CHEMBL3385 | 0.74 | 0.53 |
|  | Casein kinase I alpha | P48729 | CHEMBL2793 | 0.69 | 0.53 |
| L7 | Serine/threonine-protein kinase NEK6 | Q9HC98 | CHEMBL4309 | 0.7 | 0.62 |
|  | Dual specificity protein kinase CLK3 | P49761 | CHEMBL4226 | 0.73 | 0.58 |
|  | CaM kinase IV | Q16566 | CHEMBL2494 | 0.72 | 0.56 |
|  | Serine/threonine-protein kinase ULK2 | Q8IYT8 | CHEMBL5435 | 0.69 | 0.55 |
|  | Serine/threonine-protein kinase 11 | Q15831 | CHEMBL5606 | 0.7 | 0.55 |
| L9 | Serine/threonine-protein kinase NEK6 | Q9HC98 | CHEMBL4309 | 0.7 | 0.63 |
|  | Casein kinase I gamma 2 | P78368 | CHEMBL2543 | 0.8 | 0.62 |
|  | Receptor tyrosine-protein kinase erbB-3 | P21860 | CHEMBL5838 | 0.74 | 0.53 |
|  | Dual specificity mitogen-activated protein kinase kinase 7 | O14733 | CHEMBL3530 | 0.69 | 0.51 |
|  | Casein kinase I epsilon | P49674 | CHEMBL4937 | 0.74 | 0.48 |

Note: 2-[2-(9H-xanthen-9-yl)hydrazinyl]-1,3-dithiolan-4-one (L3), 2-[2-(9H-xanthen-9-yl)hydrazinyl]-1,3-thiazol-5(4H)-one (L5), 2-(9H-xanthen-9-ylamino)-1,3-thiazol-5(4H)-one (L7), and lower right; hydroxy(oxo)(4-{4-[(9H-xanthen-9-yloxy)methyl]-1H-1,2,3-triazol-1-yl}phenyl)ammonium (L9). AUC: area under the curve, LOO CV: leave-one-out cross-validation.

**Protein (PDB: 1YWN)-Ligand Interaction Analysis**


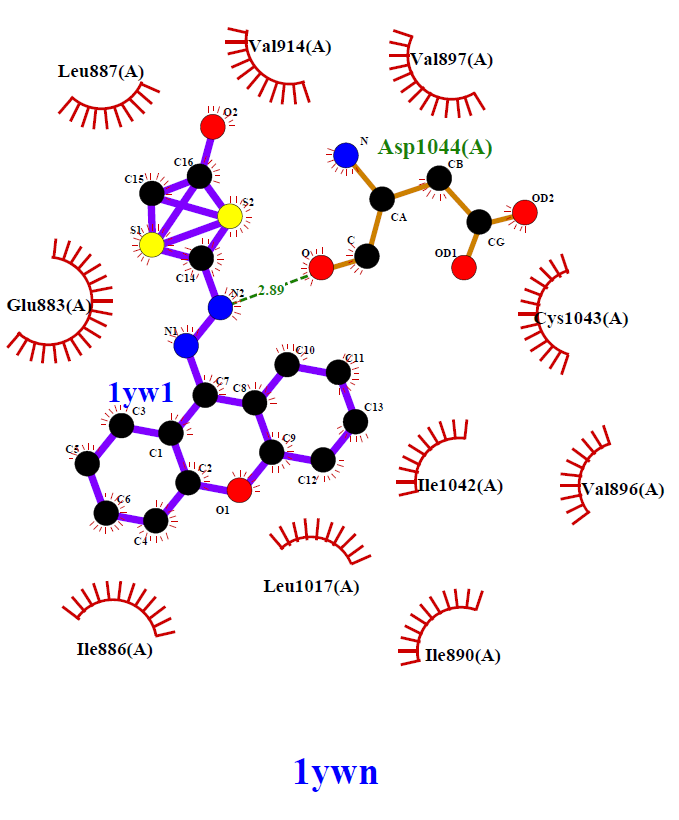

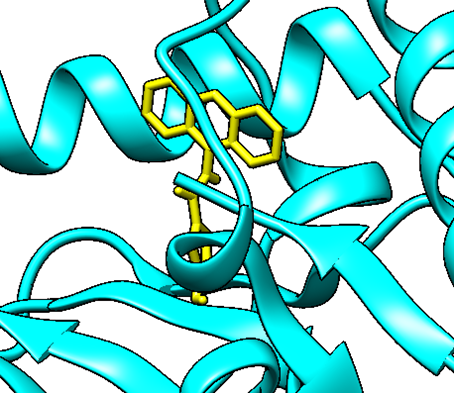


**Figure (1). 1YWN-L3 shows:** non-ligand bond. Corresponding atoms and non-ligand Ile890, Va189, Ile1042, Leu1017, Cys1043, Val1897, Val1914, Leu887, Glu883 and Ile886 involved in hydrophobic interactions. Hydrogen bonds between L3 and Asp1044 seen by dotted olive-green lines.


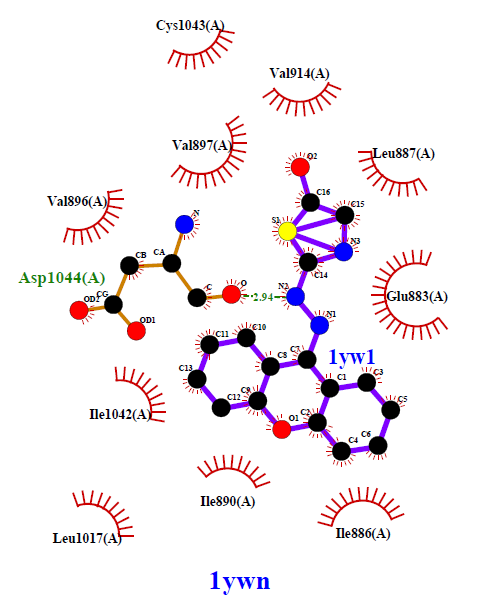

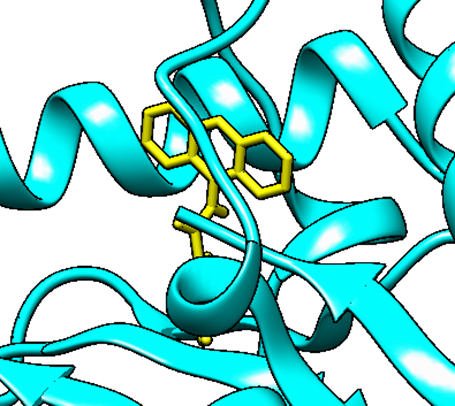


**Figure (2). 1YWN-L5 shows:** non-ligand bond. Corresponding atoms and non-ligand Leu1017, Ile890, Ile1042, Val896, Ile886, Glu883, Leu887, Val897, Val914, Cy1043 involved in hydrophobic interactions. Hydrogen bonds between L5 and Asp1044 seen by dotted olive-green lines.


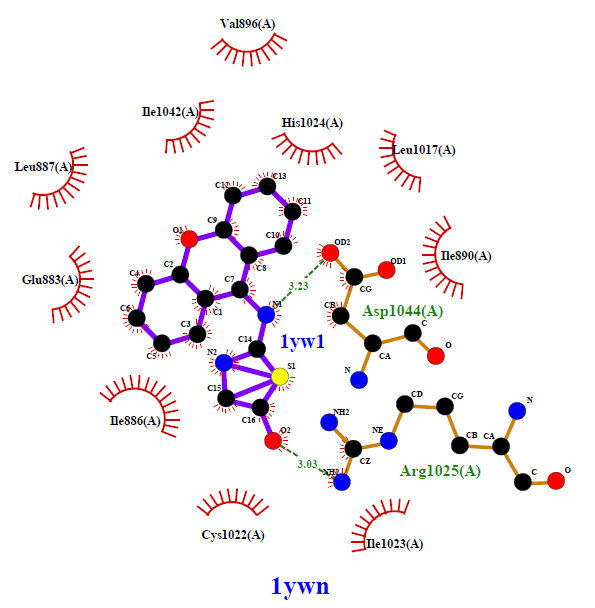

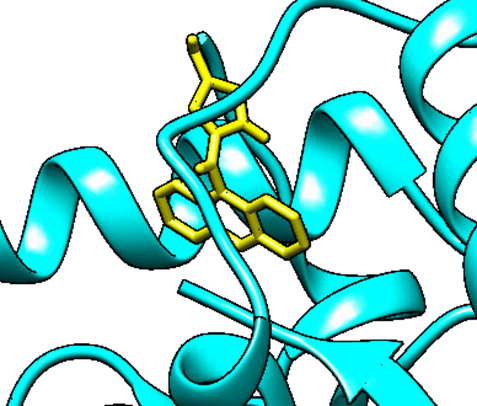


**Figure (3). 1YMN-L7** shows: non-ligand bond. Corresponding atoms and non-ligand Ile1023, Cys1022, Ile886, Glu883, Leu887, Val896, Ile1042, Leu1017, Ile890 and His1042 involved in hydrophobic interactions. Hydrogen bonds between L7 and Asp1044 and Arg1025 seen by dotted olive-green lines.


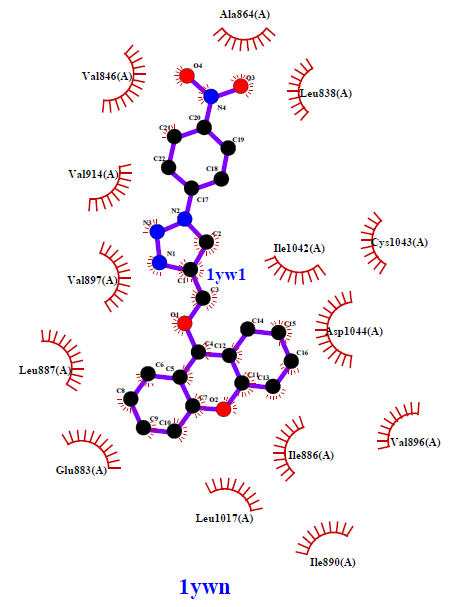

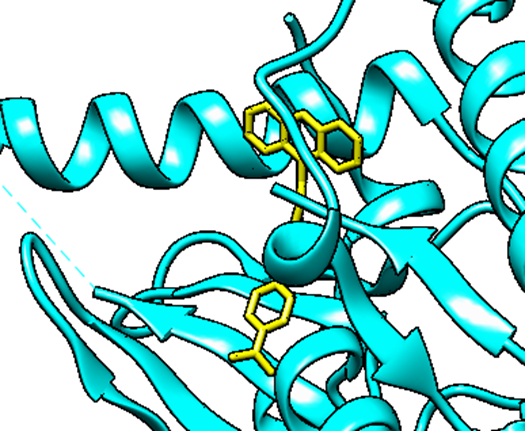


**Figure (4). 1YWN-L9 shows:** non-ligand bond. Corresponding atoms and non-ligand Ile890, Ile886, Asp1044, Ile1042, Cys1043, Leu1017, Val896, Leu887, Val897, Val846, Ala864, Leu838, Leu1033 and Val914 involved in hydrophobic interactions. Hydrogen bonds between L9 and Glu883 seen by dotted olive-green lines.

**Protein (PDB: 3LXL)-Ligand Interaction Analysis**


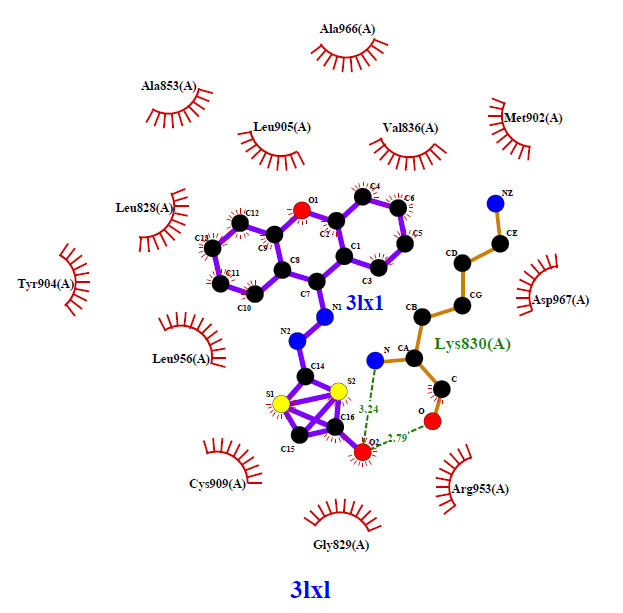

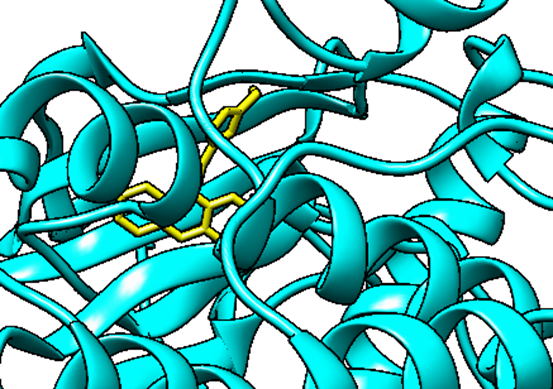


**Figure (1). 3LXL-L3 shows:** non-ligand bond. Corresponding atoms and non-ligand Gly829, Arg953, Asp967, Met902, Val836, Ala966, Leu905, Ala853, Leu828, Tyr904, Leu956, and Cys909 involved in hydrophobic interactions. Hydrogen bonds between L3 and Ly9830seen by twice dotted olive-green lines.


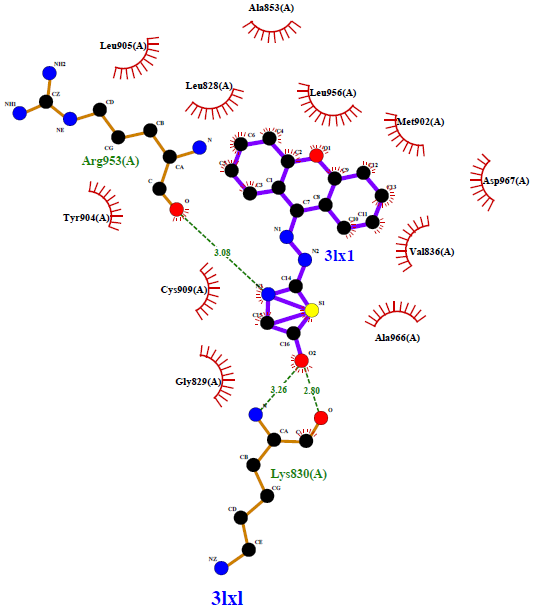

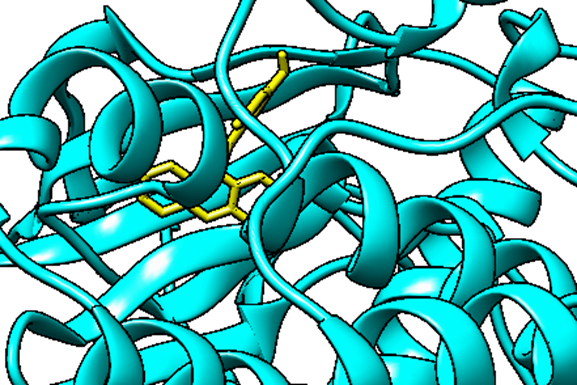


**Figure (2). 3LXL-L5 shows:** non-ligand bond. Corresponding atoms and non-ligand Ala966, Val836, Asp967, Met902, Leu956, Ala853, Leu828, Leu905, Trr904, Cys909 and Gly829 involved in hydrophobic interactions. Hydrogen bonds between L5 and Arg953 and twice with Lys830 seen by dotted olive-green lines.


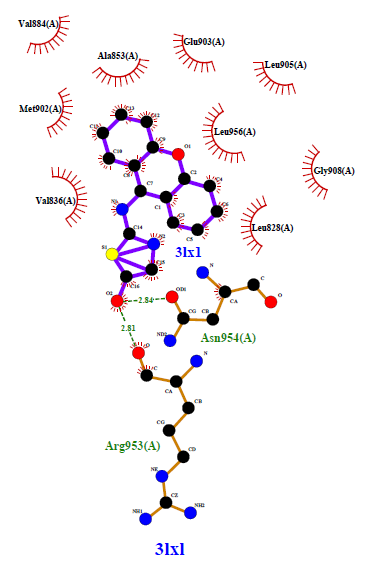

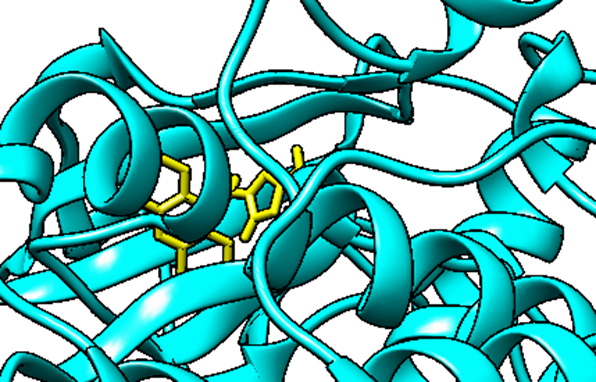


**Figure (3). 3LXL-L7 shows:** non-ligand bond. Corresponding atoms and non-ligand Leu828, Gly908, Leu956, Leu905, Glu903, Ala853, Val884, Met902 and Val836 involved in hydrophobic interactions. Hydrogen bonds between L7 and Arg953 and Asn954 seen by dotted olive-green lines.


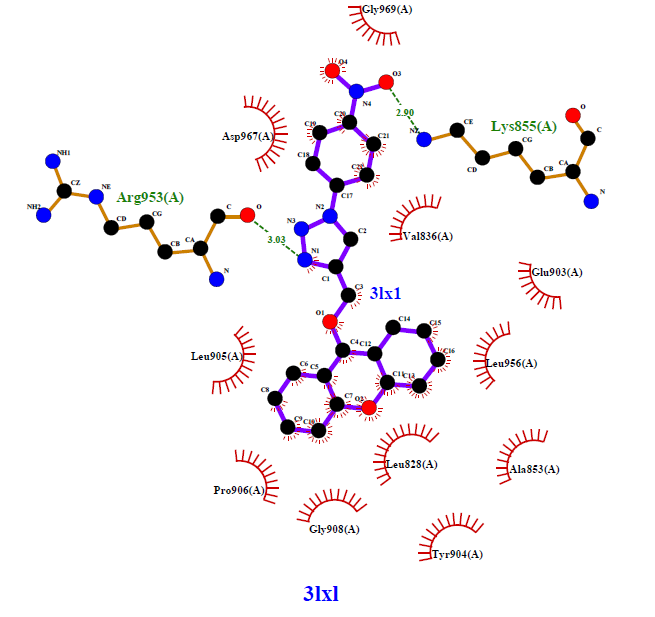

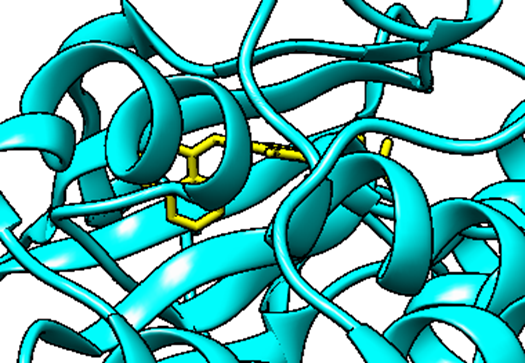


**Figure (4). 3LXL-L9 shows:** non-ligand bond. Corresponding atoms and non-ligand Tyr904, Ala853, Leu828, Gly908, Leu956, Glu903, Val836, Gly969, Asp967, Leu905 and Pro906 involved in hydrophobic interactions. Hydrogen bonds between L9 and Arg953 and Lys855 seen by dotted olive-green lines

**Protein (PDB: 5EK0)-Ligand Interaction Analysis**


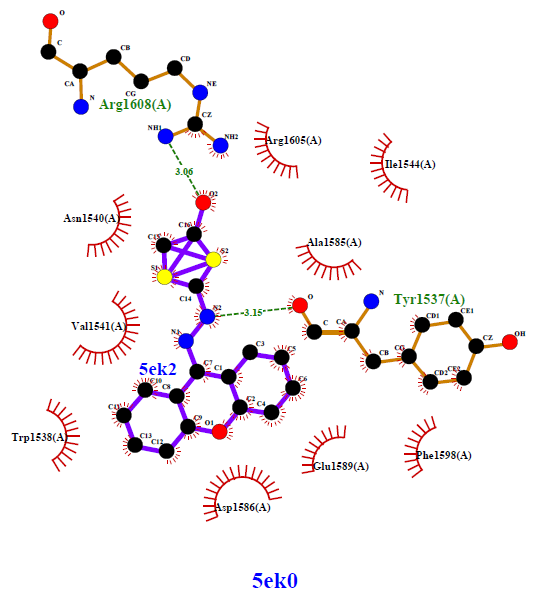

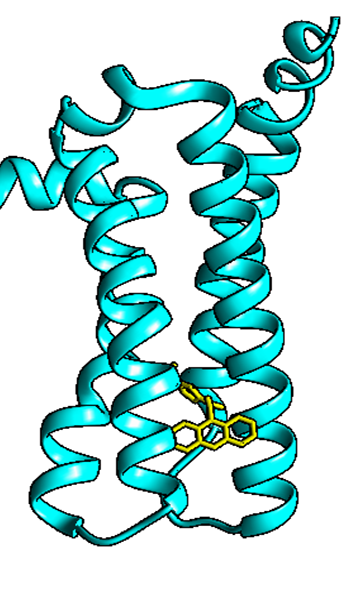


**Figure (1). 5EK0-L3 shows:** non-ligand bond. Corresponding atoms and non-ligand Phe1598, Glu1589, Asp1586, Ala1585, Ile1544, Arg1605, An1540, Val1541, and Trp1538 involved in hydrophobic interactions. Hydrogen bonds between L3 and Arg1608 and Tyr1537 seen by dotted olive-green lines.


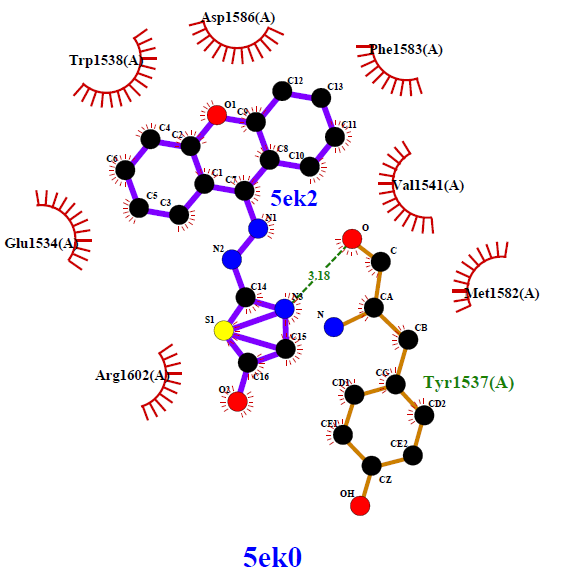

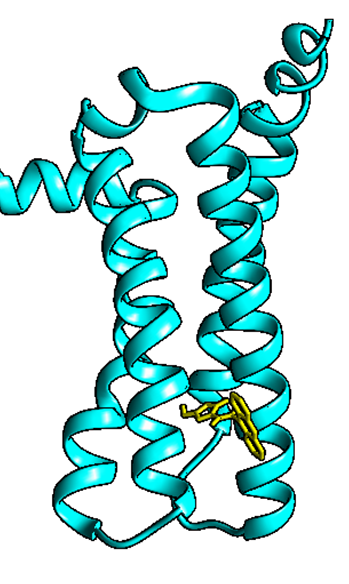


**Figure (2). 5EK0-L5 shows:** non-ligand bond. Corresponding atoms and non-ligand Met1582, Val1541, Phe1583, Asp1586, Trp1538, Glu1534 and Arg1602 involved in hydrophobic interactions. Hydrogen bonds between L5 and Tyr1537 seen by dotted olive-green lines.


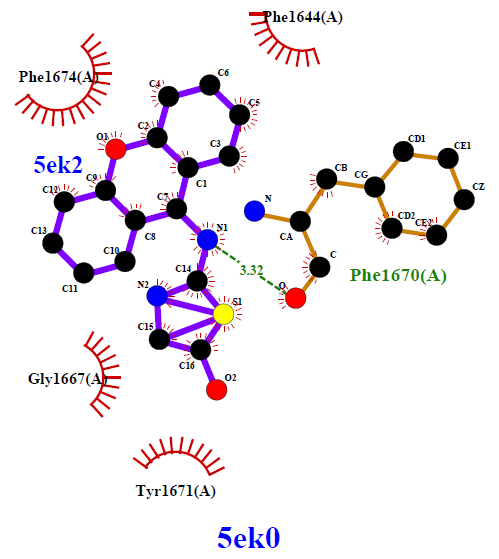

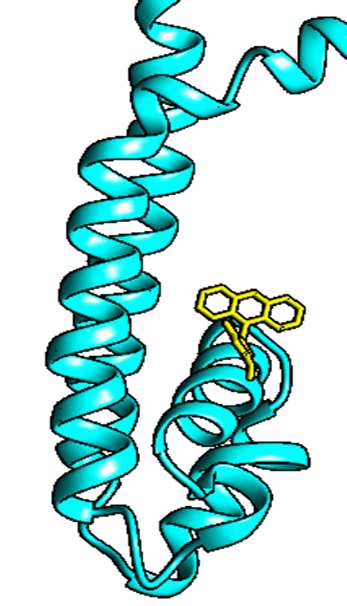


**Figure (3). 5EK0-L7 shows:** non-ligand bond. Corresponding atoms and non-ligand Tyr1671, Gly1667, Phe1644 and Phe1674 involved in hydrophobic interactions. Hydrogen bonds between L7 and Phe1670 seen by dotted olive-green lines


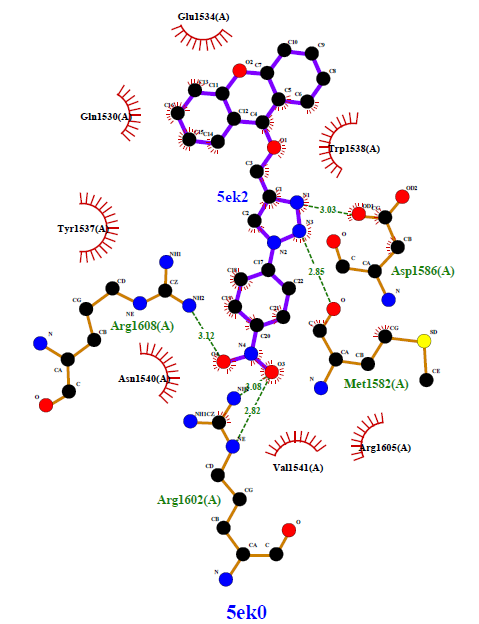

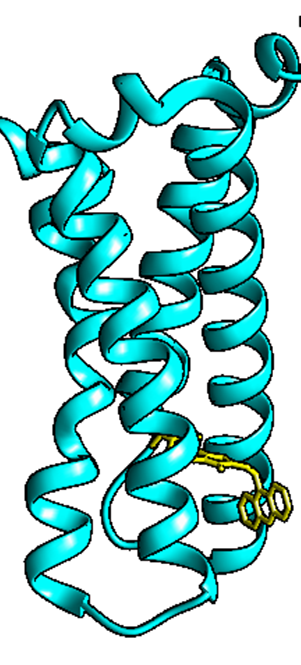


**Figure (4). 5EK0-L9 shows:** non-ligand bond. Corresponding atoms and non-ligand Val1541, Arg1605, Trp1538, Glu1534, Gln1530, Tyr1537 and Asn1540 involved in hydrophobic interactions. Hydrogen bonds between L9 and Met1582, Arg1602, Arg1608 and Asp1586 seen by dotted olive-green lines.

**Protein (PDB: 5UG9)-Ligand Interaction Analysis**


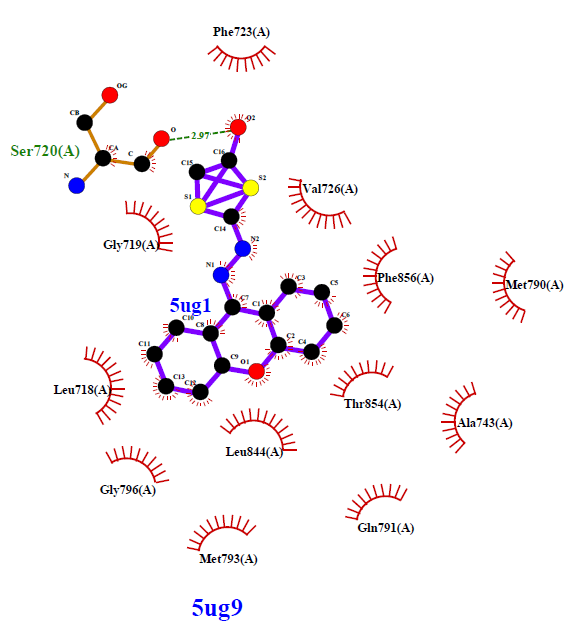

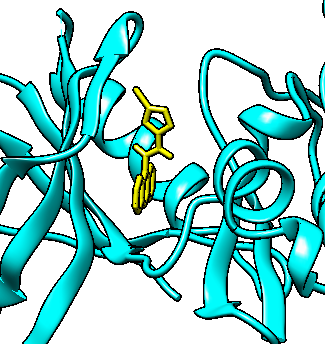


**Figure (1). 5UG9-L3 shows:** non-ligand bond. Corresponding atoms and non-ligand Met793, Gln791, Leu844, Thr854, Ala743, Phe856, Met790, Val726, Gly719, Leu718 and Gly796 involved in hydrophobic interactions. Hydrogen bonds between L3 and Ser720 seen by dotted olive-green lines.


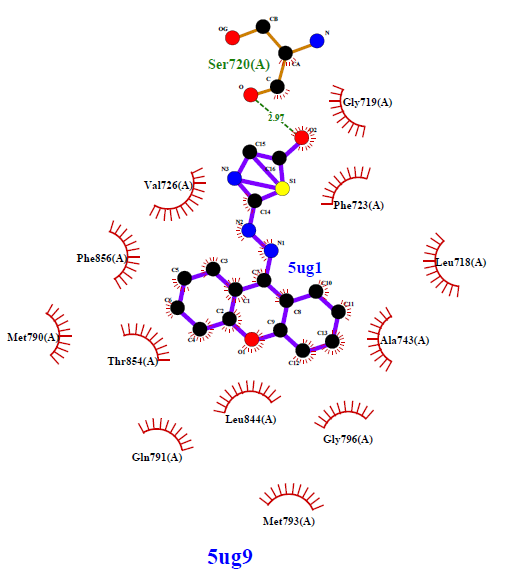

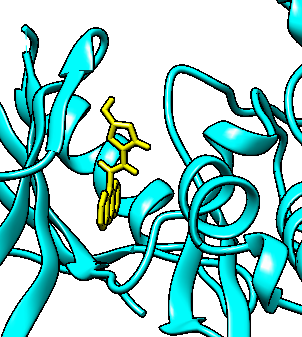


**Figure (2). 5UG9-L5 shows:** non-ligand bond. Corresponding atoms and non-ligand Met793, Gly796, Leu844, Ala742, Leu718, Phe723, Gly719, Val726, Phe856, Met790, Thr854 and Gln791 involved in hydrophobic interactions. Hydrogen bonds between L5 and Ser720 seen by dotted olive-green lines.


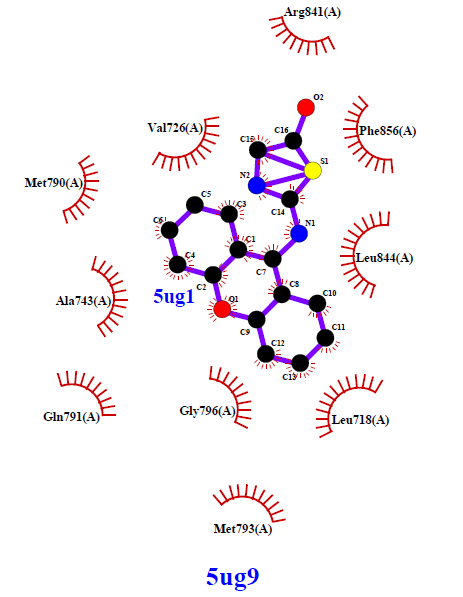

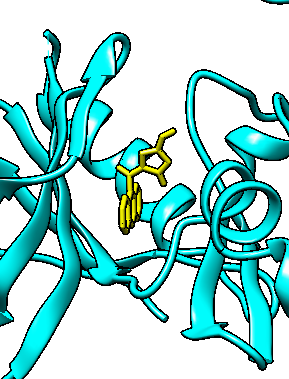


**Figure (3). 5UG9-L7 shows:** Corresponding atoms and non-ligand Met793, Gly796, Leu718, Leu844, Phe856, Arg841, Val726, Met790, Ala743, Gln791 involved in hydrophobic interactions.


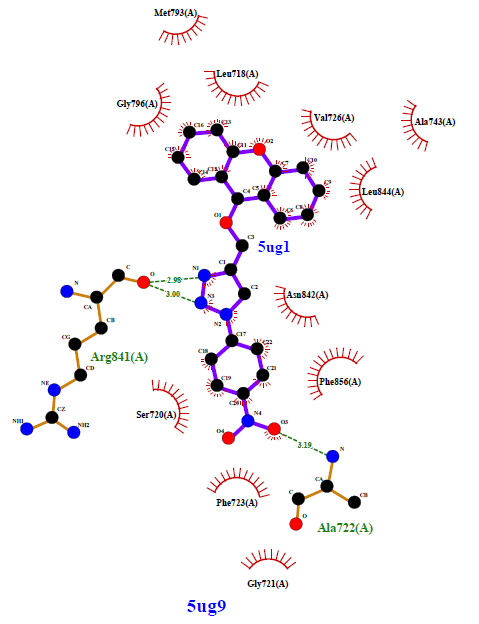

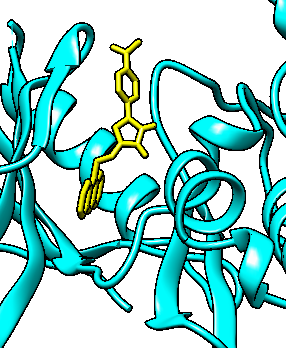


**Figure (4). 5UG9-L9 shows:** non-ligand bond. Corresponding atoms and non-ligand Phe723, Gly721, Phe856, Asn842, Leu844, Val726, Ala743, Leu718, Gly796 and Ser720 involved in hydrophobic interactions. Hydrogen bonds between L9 and Ala7222 and twice bonds with Arg841 seen by dotted olive-green lines.

**Protein (PDB: 3W8Q)-Ligand Interaction Analysis**


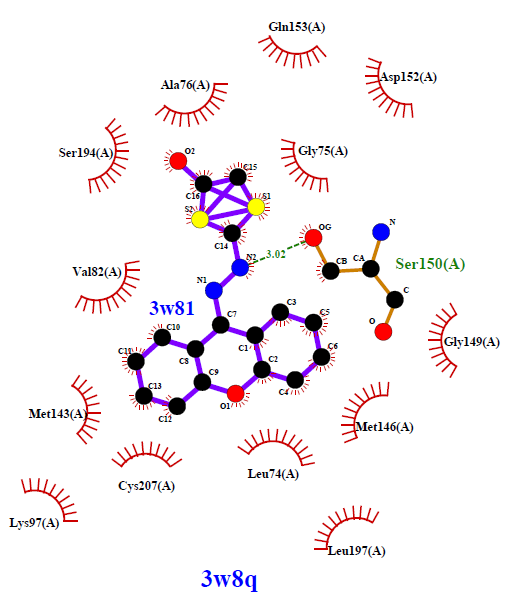

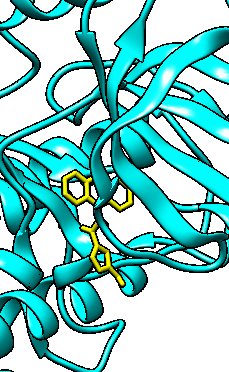


**Figure (1). 3W8Q-L3 shows:** non-ligand bond. Corresponding atoms and non-ligand Gly149, Met146, Leu197, Leu74, Cys207, Lys97, Met143, Val182, Ser194, Ala76, Gln153, Gly75 and Asp152 involved in hydrophobic interactions. Hydrogen bonds between L3 and Ser150 seen by dotted olive-green lines.


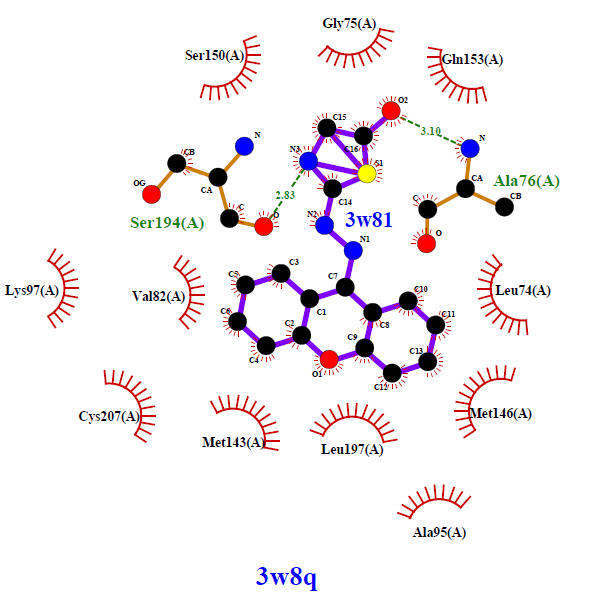

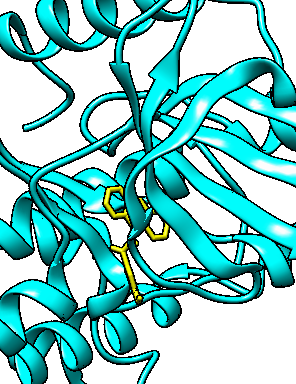


**Figure (2). 3W8Q-L5 shows:** non-ligand bond. Corresponding atoms and non-ligand Leu74, Met146, Ala95, Leu197, Met197, Met143, Cys207, Val182, Lys97, Ser150, Gly75 and Gln153 involved in hydrophobic interactions. Hydrogen bonds between L5 and Ser194 and Ala76 seen by dotted olive-green lines.


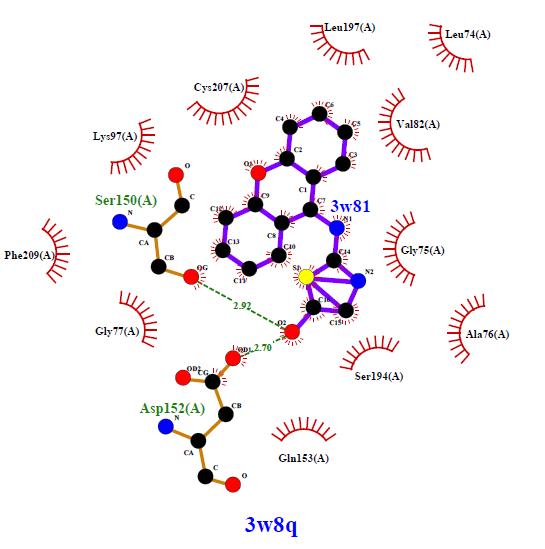

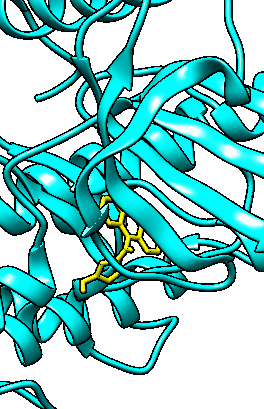


**Figure (3). 3W8Q-L7 shows:** non-ligand bond. Corresponding atoms and non-ligand Gln153, Ser194, Ala76, Gly75, Val182, Leu74, Leu197, Cys207, Lys97, Phe209 and Gly77 involved in hydrophobic interactions. Hydrogen bonds between L7 and Ser150 and Asp152 seen by dotted olive-green lines.


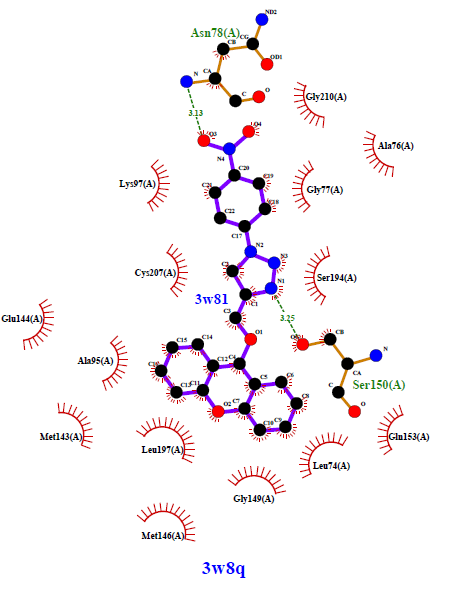

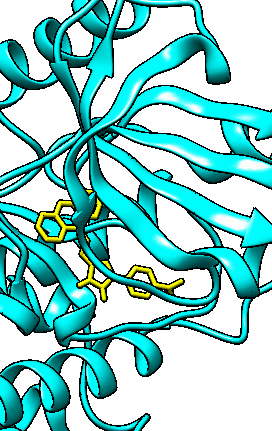


**Figure (4). 3W8Q-L9 shows:** non-ligand bond. Corresponding atoms and non-ligand Gln153, Leu74, Gly149, Met146, Leu197, Met143, Ala95, Glu144, Cys207, Lys97, Gly210, Gly77, Ser194 and Ala76 involved in hydrophobic interactions. Hydrogen bonds between L9 and Ser150 seen by dotted olive-green lines.

**Protein (PDB: 4U7Z)-Ligand Interaction Analysis**


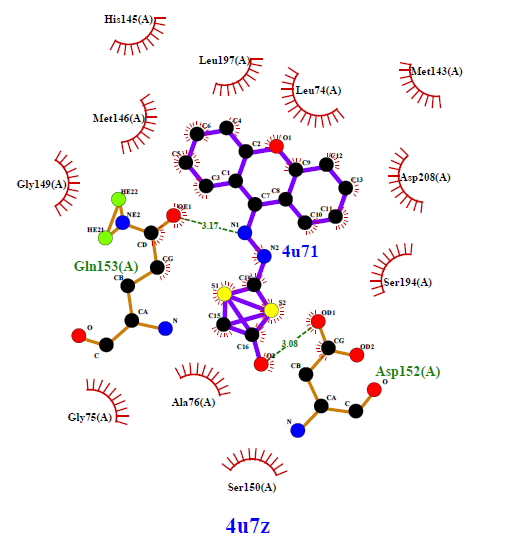

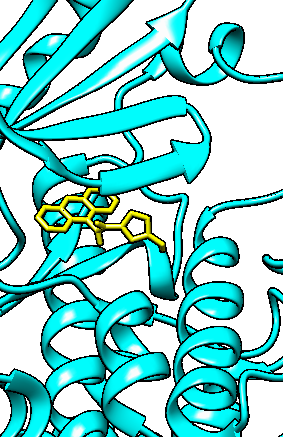


**Figure (1). 4U7Z-L3 shows:** non-ligand bond. Corresponding atoms and non-ligand Ser194, Asp208, Met143, Leu74, Leu197, His145, Met146, Gly149, Gly75, Ala76 and Ser150 involved in hydrophobic interactions. Hydrogen bonds between L3 and Gln153, Asp152 seen by dotted olive-green lines.


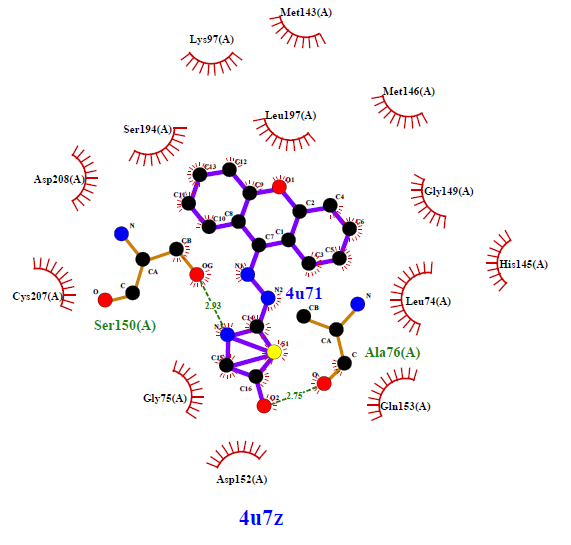

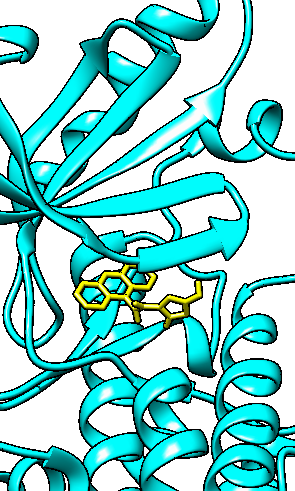


**Figure (2). 4U7Z-L5 shows:** non-ligand bond. Corresponding atoms and non-ligand Gln153, Leu74, His145, Gly149, Met145, Met143, Lys97, Leu197, Ser194, Ap208, Cy207, Gly76 and Asp152 involved in hydrophobic interactions. Hydrogen bonds between L5 and Ala76, Ser150 seen by dotted olive-green lines.


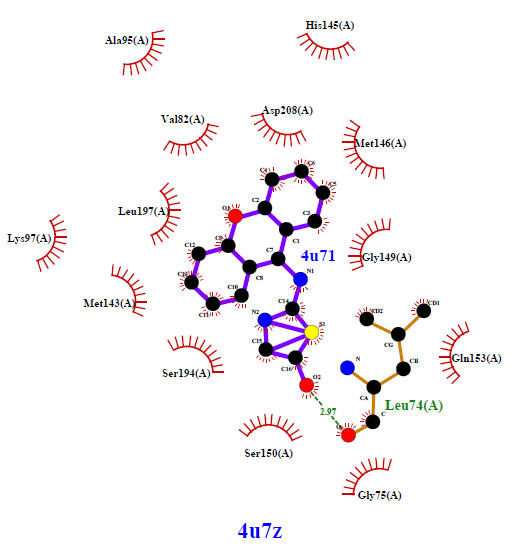

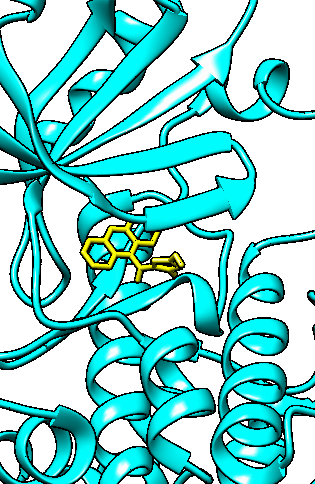


**Figure (3). 4U7Z-L7 shows:** non-ligand bond. Corresponding atoms and non-ligand Gly149, Met146, Asp208, Val182, His145, Ala95, Leu197, Lys97, Met143, Ser194, Ser150, Gly75 and Gln153 involved in hydrophobic interactions. Hydrogen bonds between L7 and Leu74 seen by dotted olive-green lines.


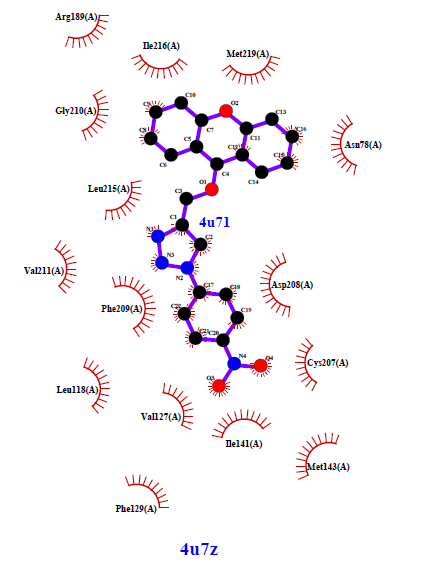

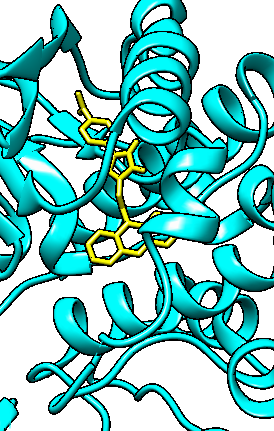


**Figure (4). 4U7Z-L9 shows:** Corresponding atoms and non-ligand Asp208, Cys207, Met143, Ile141, Val127, Phe129, Leu118, Phe209, Phe129, Val121, Leu215, Gly210, Arg189, Ile216, Met219, Asn78 involved in hydrophobic interactions.
