# Supplementary material for: Synthesis, characterization, and computational evaluation of some synthesized xanthone derivatives: focus on kinase target network and biomedical properties
Source: Front Pharmacol. 2025 Jan 3;15:1511627. doi: 10.3389/fphar.2024.1511627 (PMC11738930; doi:10.3389/fphar.2024.1511627)
Supplement: Supplementary file 1 [file DataSheet1.zip › Supplementary file 8.DOCX]

**Synthesis, Characterization, and Computational Evaluation of Some Synthesized Xanthone Derivatives: Focus on Kinase Target Network and Biomedical Properties**

**Wisam Taher Muslim^1^, Layth Jasim Mohammad^2¥^, Munaf M. Naji^3^, Isaac Karimi^4,5^*^¥^, Matheel D. Al-Sabti^56^, Majid Jabir^7^, Mazin A. A. Najm^8^, Helgi B. Schiöth^5^*^¥^**

^1^Department of Pharmaceutical Chemistry, College of Pharmacy, Kufa University, Najaf City, Najaf Governorate, 540011, Iraq.

^2^ Department of Microbiology, College of Medicine, Babylon University, Hilla City, Babylon Governorate, 51002, Iraq.

^3^Clinical-Laboratory Sciences, College of Pharmacy, Kufa University, Najaf City, Najaf Governorate, 540011, Iraq.

^4*^Reseach Group of Bioengineering and Biotechnology, Laboratory for Computational Physiology; Department of Biology, Faculty of Science, Razi University 67149-67346, Kermanshah, Iran.

^5*^Department of Surgical Sciences, Functional Pharmacology and Neuroscience, Uppsala University, 751 24, Uppsala, Sweden.

^6^Department of Science, College of Science, Uruk University, Baghdad, Iraq.

^7^Department of Applied Science, University of Technology, Baghdad, Iraq.

^8^Department of Pharmacy, Mazaya University Collage, Nasiriyah, Thi-Qar, Iraq.

^¥^These authors contributed equally to this work

**Corresponding authors**: Helgi B. Schiöth, [helgi.schioth@uu.se](mailto:helgi.schioth@uu.se), Tel and Fax: 0046-18-4714160; Isaac Karimi; [isaac-karimi2000@yahoo.com](mailto:isaac-karimi2000@yahoo.com); [karimiisaac@razi.ac.ir](mailto:karimiisaac@razi.ac.ir). Tel & Fax: 0098-83-34274545.

**Table 1.** Determining the network weights of kinase targets of xanthone derivatives

| **Gene symbol** | **Score** | **Functions/link** |
| --- | --- | --- |
| HASPIN | 0.8295 | Chromosome segregation, histone kinase activity, histone phosphorylation, mitotic nuclear division, nuclear chromosome segregation, peptidyl-threonine modification, peptidyl-threonine phosphorylation, protein localization to chromosome, centromeric region, and protein serine/threonine kinase activity.  <http://www.ncbi.nlm.nih.gov/sites/entrez?db=gene&cmd=search&term=83903> |
| WEE2 | 0.8080 | Oocyte meiosis inhibiting kinase, cell cycle checkpoint, negative regulation of mitotic cell cycle, nucleus organization, and regulation of nuclear division.  <http://www.ncbi.nlm.nih.gov/sites/entrez?db=gene&cmd=search&term=494551> |
| PIM3 | 0.8028 | Carbohydrate homeostasis, glucose homeostasis, cellular response to carbohydrate, glucose, hexose, and monosaccharide stimuli, glucose homeostasis, and insulin and peptide hormone secretion/regulation.  <http://www.ncbi.nlm.nih.gov/sites/entrez?db=gene&cmd=search&term=415116> |

**Table 2**. *In silico* prediction of the antitarget profile of xanthone derivatives.

|  |  | Ligands (Prediction value, -log10(value), mole) | | | |
| --- | --- | --- | --- | --- | --- |
|  | Endpoint | **L3** | **L5** | **L7** | **L9** |
| 5-hydroxytryptamine 1B receptor antagonist | IC50 | 5,078* | 5,447 | 5,113 | 6,043* |
| 5-hydroxytryptamine 1B receptor antagonist | Ki | 6,316 | 6,308 | 6,184 | 6,588 |
| 5-hydroxytryptamine 2A receptor antagonist | IC50 | 7,109* | 6,966* | 6,842* | 5,754 |
| 5-hydroxytryptamine 2A receptor antagonist | Ki | 6,192* | 6,936 | 6,970 | 6,705 |
| 5-hydroxytryptamine 2C receptor antagonist | IC50 | 7,706 | 7,374 | 7,326 | 5,661 |
| 5-hydroxytryptamine 2C receptor antagonist | Ki | 6,846 | 7,373 | 7,394 | 6,570 |
| alpha1a adrenergic receptor antagonist | IC50 | 5,863* | 5,660 | 5,998 | 5,796 |
| alpha1a adrenergic receptor antagonist | Ki | 6,013* | 5,120 | 4,999 | 5,631* |
| alpha1b adrenergic receptor antagonist | Ki | 6,668 | 7,016 | 6,875 | 6,911 |
| alpha-2A adrenergic receptor antagonist | IC50 | 6,190 | 5,418 | 5,448 | 6,947 |
| alpha-2A adrenergic receptor antagonist | Ki | 6,262 | 5,994 | 6,439 | 6,237 |
| amine oxidase [flavin-containing] A inhibitor | IC50 | 5,505 | 5,726 | 5,651 | 5,729 |
| amine oxidase [flavin-containing] A inhibitor | Ki | 5,199 | 5,359 | 5,555 | 6,562 |
| androgen receptor antagonist | IC50 | 5,736 | 5,618 | 5,769 | 6,276 |
| carbonic anhydrase II activator | Kact | 7,847 | 7,425 | 8,052 | 8,172 |
| carbonic anhydrase I activator | Kact | 6,399 | 7,101 | 8,206 | 7,278 |
| carbonic anhydrase I inhibitor | Ki | 5,649 | 6,650 | 6,676 | 6,854 |
| carbonic anhydrase II inhibitor | IC50 | 7,924 | 6,737* | 7,794* | 8,709 |
| d(1A) dopamine receptor antagonist | IC50 | 5,295 | 4,945 | 4,908 | 5,779 |
| d(1A) dopamine receptor antagonist | Ki | 6,378 | 5,708 | 5,614 | 5,347 |
| d3 dopamine receptor antagonist | Ki | 7,458* | 6,705* | 5,903* | 6,702* |
| delta-type opioid receptor antagonist | Ki | 5,706* | 6,354* | 6,431* | 6,077* |
| estrogen receptor antagonist | IC50 | 6,002 | 5,624 | 5,887 | 5,521 |
| estrogen receptor antagonist | Ki | 5,910 | 5,723 | 5,810 | 6,248 |
| kappa-type opioid receptor antagonist | Ki | 6,177* | 6,513* | 6,134* | 7,025* |
| mu-type opioid receptor antagonist | IC50 | 7,228 | 5,472* | 6,139 | 5,742 |
| mu-type opioid receptor antagonist | Ki | 6,650* | 5,578* | 6,850* | 5,282* |
| sodium- and chloride-dependent GABA transporter 1 antagonist | IC50 | 4,915 | 3,845 | 4,525 | 6,363 |
| sodium-dependent dopamine transporter antagonist | IC50 | 5,606* | 5,879 | 5,705 | 6,339 |
| sodium-dependent dopamine transporter antagonist | Ki | 6,025* | 5,825 | 6,150 | 6,261 |
| sodium-dependent serotonin transporter antagonist | IC50 | 5,722 | 5,563 | 5,648 | 6,164 |
| sodium-dependent serotonin transporter antagonist | Ki | 6,974* | 6,469* | 5,813* | 6,493* |

Note: * Out of applicability domain; 2-[2-(9H-xanthen-9-yl)hydrazinyl]-1,3-dithiolan-4-one (L3), 2-[2-(9H-xanthen-9-yl)hydrazinyl]-1,3-thiazol-5(4H)-one (L5), 2-(9H-xanthen-9-ylamino)-1,3-thiazol-5(4H)-one (L7), and lower right; hydroxy(oxo)(4-{4-[(9H-xanthen-9-yloxy)methyl]-1H-1,2,3-triazol-1-yl}phenyl)ammonium (L9). 50% inhibitory concentration - IC_50,_ inhibition constant - K_i_, and activation constant - K_act_

**Table 3.** The prediction of tissue toxicity of xanthone derivatives using ADVER-Pred

|  | L3 | L5 | L7 | L9 |
| --- | --- | --- | --- | --- |
| Hepatotoxicity | 0.607 | 0.590 | 0.378 | 0.535 |
| Cardiac failure |  |  | 0.487 |  |
| Myocardial infarction |  |  | 0.463 |  |
| Arrhythmia |  |  | 0.440 |  |

Note: 2-[2-(9H-xanthen-9-yl)hydrazinyl]-1,3-dithiolan-4-one (L3), 2-[2-(9H-xanthen-9-yl)hydrazinyl]-1,3-thiazol-5(4H)-one (L5), 2-(9H-xanthen-9-ylamino)-1,3-thiazol-5(4H)-one (L7), and lower right; hydroxy(oxo)(4-{4-[(9H-xanthen-9-yloxy)methyl]-1H-1,2,3-triazol-1-yl}phenyl)ammonium (L9).

**Table 4*.*** *In silico* cell line cytotoxicity prediction of xanthone derivatives

| Compound | Cell line | Cell line name | Target/organ | Pa | Pi |
| --- | --- | --- | --- | --- | --- |
| L3 | HEK293 | Embryonic kidney fibroblast | Kidney | 0.224 | 0.089 |
|  | MRC5 | Embryonic lung fibroblast | Lung | 0.229 | 0.102 |
|  | CRL-7065 | Fibroblast | Skin | 0.117 | 0.015 |
|  | PrEC | Prostate epithelial cell | Prostate | 0.145 | 0.073 |
|  | WIL2-NS | Lymphoblastoid cell | Hematopoietic, lymphoid tissue | 0.079 | 0.031 |
|  | AG1523 | Fibroblast | Fibroblast | 0.051 | 0.014 |
|  | MT2 | Lymphocyte (HTLV-1 producing cell line) | Blood | 0.083 | 0.047 |
|  | TERT-RPE1 | Retinal pigmented epithelial cell | Retina | 0.039 | 0.014 |
| L5 | MRC5 | Embryonic lung fibroblast | Lung | 0.234 | 0.098 |
|  | PrEC | Prostate epithelial cell | Prostate | 0.133 | 0.102 |
|  | AG1523 | Fibroblast | Fibroblast | 0.048 | 0.019 |
|  | IMR-90 | Embryonic lung fibroblast | Lung | 0.136 | 0.128 |
|  | TERT-RPE1 | Retinal pigmented epithelial cell | Retina | 0.033 | 0.026 |
| L7 | PrEC | Prostate epithelial cell | Prostate | 0.127 | 0.120 |
| L9 | HEL 299 | Fibroblast | Lung | 0.163 | 0.105 |
|  | WIL2-NS | Lymphoblastoid cell | Hematopoietic, lymphoid tissue | 0.072 | 0.047 |

Note: Pa: Probable activity, Pi: Probable inactivity. 2-[2-(9H-xanthen-9-yl)hydrazinyl]-1,3-dithiolan-4-one (L3), 2-[2-(9H-xanthen-9-yl)hydrazinyl]-1,3-thiazol-5(4H)-one (L5), 2-(9H-xanthen-9-ylamino)-1,3-thiazol-5(4H)-one (L7), and lower right; hydroxy(oxo)(4-{4-[(9H-xanthen-9-yloxy)methyl]-1H-1,2,3-triazol-1-yl}phenyl)ammonium (L9).

**Table 5*.*** *In silico* cancer cell line cytotoxicity prediction of xanthone derivatives

| Compound | Cell line | Target tissue | Tumor type | Pa | Pi |
| --- | --- | --- | --- | --- | --- |
| L3 | HeLa | Cervix | Cervical adenocarcinoma | 0.446 | 0.032 |
|  | T98G | Brain | Glioblastoma | 0.374 | 0.021 |
|  | Hs 683 | Brain | Oligodendroglioma | 0.428 | 0.098 |
|  | MKN-7 | Stomach | Gastric carcinoma | 0.250 | 0.110 |
| L5 | Jurkat | Blood | Acute leukemic T-cells | 0.673 | 0.004 |
|  | T98G | Brain | Glioblastoma | 0.377 | 0.020 |
|  | Hs 683 | Brain | Oligodendroglioma | 0.425 | 0.100 |
|  | 5637 | Urinary tract | Urothelial bladder carcinoma | 0.245 | 0.223 |
| L7 | Jurkat | Blood | Acute leukemic T-cells | 0.789 | 0.004 |
|  | T98G | Brain | Glioblastoma | 0.370 | 0.024 |
|  | Hs 683 | Brain | Oligodendroglioma | 0.404 | 0.114 |
|  | CCRF-CEM | Blood | Childhood T acute lymphoblastic leukemia | 0.239 | 0.092 |
| L9 | HuP-T3 | Pancreas | Pancreatic adenocarcinoma | 0.358 | 0.021 |
|  | MDA-MB-231 | Breast | Breast adenocarcinoma | 0.352 | 0.065 |
|  | HCT-15 | Colon | Colon adenocarcinoma | 0.315 | 0.066 |
|  | SAOS-2 | Bone | Osteosarcoma | 0.261 | 0.023 |

Note: Pa: Probable activity, Pi: Probable inactivity. 2-[2-(9H-xanthen-9-yl)hydrazinyl]-1,3-dithiolan-4-one (L3), 2-[2-(9H-xanthen-9-yl)hydrazinyl]-1,3-thiazol-5(4H)-one (L5), 2-(9H-xanthen-9-ylamino)-1,3-thiazol-5(4H)-one (L7), and lower right; hydroxy(oxo)(4-{4-[(9H-xanthen-9-yloxy)methyl]-1H-1,2,3-triazol-1-yl}phenyl)ammonium (L9).
